# Extracellular vesicles from glycolytic mesenchymal stromal cells restrain arthritis progression via IL-10–Producing T and B cells

**DOI:** 10.7150/thno.123898

**Published:** 2026-05-11

**Authors:** Eliana Lara-Barba, Raúl Lagos, Yesenia Flores-Elías, Yeimi Herrera-Luna, Noymar Luque-Campos, María Jesús Araya-Sapag, Constanza Aros-Valdivia, Felipe A. Bustamante-Barrientos, Liliana Yantén-Fuentes, Consuelo Covarruvias-Segovia, Consuelo Contreras, María Ignacia Cádiz, César Merino-Flores, Grégory Collin, Yessia Hidalgo-Fadic, Aliosha I Figueroa-Valdés, Hugo Tobar, Francisca Alcayaga-Miranda, María Paz Hernandez, Gino Nardocci, Estefanía Nova-Lamperti, Karine Toupet, Carolina Pradenas-Fuenzalida, Claudia Terraza, Francisca Uribe, Andrés Villarroel, Jasna V. Campos, Marcela Mondaca, Pablo Cruz, Andrea Matamoros, Alvaro A. Elorza, Claudio Carril, Carlos Farkas, Karina Oyarce, Andy J Pérez, Ana María Vega-Letter, Roberto Elizondo-Vega, Farida Djouad, Patricia Luz-Crawford

**Affiliations:** 1 Centro de Investigación e Innovación Biomédica (CIIB), Facultad de Medicina, Universidad de los Andes, Santiago, Chile.; 2 IMPACT, Center of Interventional Medicine for Precision and Advanced Cellular Therapy, Santiago, Chile.; 3 Laboratorio de Biología Celular, Departamento de Biología Celular, Facultad de Ciencias Biológicas, Universidad de Concepción, Chile.; 4 IRMB, Université de Montpellier, INSERM, Montpellier, France.; 5 Cells for Cells and Regenero the chilean consortium for regenerative medicine, Santiago, Chile.; 6 Laboratorio de Neuroinmunología, Departamento de Bioquímica Clínica e Inmunología, Facultad de Farmacia, Universidad de Concepción, Concepción, Chile.; 7 Laboratorio de Investigación en Ciencias Biomédicas, Departamento de Ciencias Básicas y Morfología, Facultad de Medicina, Universidad Católica de la Santísima Concepción, Concepción, Chile.; 8 Departamento de Análisis Instrumental, Facultad de Farmacia, Universidad de Concepción, Concepción, Chile.; 9 Escuela de Ingeniería Bioquímica, Pontificia Universidad Católica de Valparaíso, Valparaíso, Chile.; 10 Escuela de Kinesiología, Facultad de Medicina, Universidad de los Andes, Santiago, Chile.; 11 Escuela de Tecnología Médica, Facultad de Medicina, Universidad de Chile, Santiago, Chile.; 12 Institute of Biomedical Sciences, Faculty of Medicine, Universidad Andres Bello Santiago, Chile.; 13 Departamento de Ciencias Biológicas y Quimicas, Facultad de Ciencias, Universidad San Sebastián, Sede Concepción, Chile.; 14 Molecular and Translational Immunology Laboratory, Department of Clinical Biochemistry and Immunology, Pharmacy Faculty, Universidad de Concepción, Concepción, Chile.

**Keywords:** EVs, glycolytic UC-MSC, rheumatoid arthritis

## Abstract

Mesenchymal stromal cells (MSCs) exert potent immunomodulatory effects largely mediated by extracellular vesicles (EVs). We previously demonstrated that glycolytic reprogramming enhances the immunosuppressive capacity of human umbilical cord–derived MSCs (UC-MSCs). Here, we investigated whether this enhanced activity is transmitted through EVs and explored the contribution of EV-associated microRNAs.

**Methods:**

EVs from naïve and glycolytically reprogrammed UC-MSCs (EVs-UC-MSC_naive_ and EVs-UC-MSC_glyco_) were isolated, characterized, and tested for their effects on memory CD4⁺ T and B cells *in vitro* and their therapeutic efficacy *in vivo* in the delayed-type hypersensitivity (DTH) and in the collagen induced arthrtis (CIA) murine model.

**Results:**

EVs-UC-MSC_glyco_ more effectively suppressed inflammatory T cell responses, promoted IL-10–producing Tr1 and B cells, and enhanced B cell survival compared with EVs-UC-MSC_naive_.* In vivo*, EVs-UC-MSC_glyco_ significantly reduced inflammation in a DTH murine model and decreased arthritis incidence and clinical severity in CIA. These effects were associated with increased Treg/Th1, Treg/Th17, Tr1/Th1, and Tr1/Th17 ratios, together with enhanced IL-10 production. MicroRNA profiling revealed enrichment of regulatory miRNAs, including miR-365a-5p, linked to suppression of pro-inflammatory signaling and activation of the IL-10 regulatory axis.

**Conclusions:**

Glycolytic reprogramming enhances the therapeutic potential of UC-MSC-derived EVs, highlighting EVs-UC-MSC_glyco_ as promising immunomodulatory candidates for the treatment of autoimmune diseases such as arthritis.

## 1. Introduction

Autoimmune diseases like rheumatoid arthritis (RA) result from a loss of immune tolerance, largely driven by imbalances among B and CD4^+^ T cell subsets. Pro-inflammatory Th17 and Th1 cells contribute to disease by promoting chronic inflammation and tissue damage through cytokines like IL-17, IFN-γ, and TNF-α. In contrast, regulatory T cells (Tregs), which mainly produce IL-10, counteract inflammation and maintain immune homeostasis. A shift favoring Th17/Th1 over Tregs is a key feature of RA, leading to uncontrolled inflammation and joint damage^1^. These insights underscore the need for therapeutic strategies that restore the Th1/Th17/Treg balance to mitigate disease severity and regulate immune dysfunction.

Current RA treatments focus on reducing inflammation, but many patients face treatment resistance or side effects^2^. MSCs have emerged as a promising alternative due to their ability to suppress pro-inflammatory Th1 and Th17 cells while promoting B and T regulatory cells, helping to restore immune balance. This mechanism is particularly relevant in RA, where Th1/Th17-driven inflammation contributes to joint damage. Additionally, MSCs support tissue repair and induce long-lasting immunological changes**,** offering multifaceted therapeutic benefits. However, their clinical use is limited by inconsistent therapeutic outcomes, often influenced by donor and tissue-specific factors.

Recently, we demonstrated that pharmacologically inducing glycolytic metabolism in MSC using oligomycin enhances their immunoregulatory and anti-inflammatory properties both *in vitro* and *in vivo^3^,^4^*. Moreover, paracrine factors secreted by glycolytically reprogrammed umbilical cord-derived MSC (UC-MSCs), mediate their enhanced therapeutic activity. These glycolytic UC-MSCs suppress proinflammatory cell responses and promote Treg function in peripheral blood mononuclear cells (PBMCs) isolated from RA patients (Luque et al, unpublish data).

Among these paracrine factors, extracellular vesicles (EVs) are key mediators of MSCs therapy, including in RA^5^-^7^. These small, membrane-bound particles (50–200 nm) facilitate intercellular communication by transferring bioactive molecules such as proteins, lipids, metabolites, and nucleic acids to target cells. In RA, MSC-derived EVs have shown therapeutic potential by modulating immune responses, reducing oxidative stress, and preventing cell death^8^,^9^. Notably, their microRNA cargo plays a central role in suppressing proinflammatory T cell activity and reprogramming immune responses toward an anti-inflammatory state, positioning EVs as a promising mechanism-based therapy for autoimmune conditions like RA^7^,^10^,^11^.

Here, we investigated the immunoregulatory and therapeutic potential of EVs derived from UC-MSCs that were metabolically primed toward glycolysis. We hypothesized that glycolytic UC-MSC-derived EVs exhibit enhanced immunoregulatory capacity compared to EVs from control UC-MSCs. Specifically, we assessed their effect on Th1 and Th17 cell differentiation and proliferation, B cell viability and IL-10 production, and Treg induction. Their therapeutic potential was evaluated using two murine models: the inflammatory delayed-type hypersensitivity (DTH) model and the autoimmune rheumatoid arthritis (RA) model, followed by microRNA profiling. These results support their development as a refined, acellular therapeutic approach for autoimmune diseases such as RA, potentially avoiding complications associated with direct MSC transplantation, including immune rejection, variable cell engraftment, and formation of cellular debris.

## 2. Materials and Methods

### 2.1. Isolation and characterization of EVs-UC-MSC

UC-MSCs at passages 5–6 were used as single donors and isolated from healthy term pregnancies obtained from four different women undergoing cesarean section, with no pregnancy-related pathologies. Umbilical cords were collected under GMP conditions at Clínica Universidad de los Andes from male newborns. Then, they were cultured in cell factories using high-glucose Dulbecco's Modified Eagle Medium (DMEM; Gibco, Thermo Fisher, USA) supplemented with 5% human platelet lysate (hPL), 1% penicillin/streptomycin, and 1% glutamine (Gibco, Thermo Fisher, USA), at 37°C and 5% CO₂ under normoxic conditions. At approximately 80% confluence, cells were either left untreated or stimulated for 24 hours with 1 µg/mL oligomycin (Tocris Bioscience, UK). Following stimulation, cells were washed three times with PBS, and the medium was replaced with serum-free DMEM. After 48 hours, the culture supernatants were collected and processed for EV isolation.

Supernatants were first centrifuged at 600 × g for 10 minutes, followed by 2,000 × g for 10 minutes at 4°C to remove cells and debris. The clarified supernatant was then filtered sequentially through 0.45 µm and 0.22 µm membranes to eliminate larger vesicles and protein aggregates. EVs were isolated by ultracentrifugation at 100,000 × g for 60 minutes using a horizontal rotor (ThermoFisher, TH641, Eq.41395256). The EV pellet was resuspended in PBS, subjected to a second ultracentrifugation under the same conditions, and the final EVs were stored at -80°C until use.

EV characterization was performed in accordance with the “Minimal Information for Studies of Extracellular Vesicles” (MISEV) guidelines^12^. EV concentration, size, and distribution were determined using Nanoparticle Tracking Analysis (NTA; Nanosight NS300, Malvern Instruments, UK), with camera level set to 8, and threshold set to 3. For surface marker profiling, 1.4 × 10⁹ EVs particles were bound to aldehyde/sulfate latex microspheres (Cat. #A37304, Life Technologies) and incubated with antibodies against human CD63 (Cat. #556019), CD81 (Cat. #555675), and CD9 (Cat. #555370; all from BD Pharmingen). Western blotting was used to detect Syntenin-1, Tomm20, Calnexin, and Flotillin-1. The presence of residual oligomycin in EVs preparations was assessed via LC-MS analysis (see Supplementary Materials). Additionally, EV internalization was evaluated by confocal microscopy, and flow cytometry.

### 2.2. Seahorse XF OCR/ECAR analysis

Cells were seeded in XF microplates at 10.000 cells/well. On the day of the assay the medium was replaced with Seahorse XF assay medium (unbuffered non glucose DMEM-based medium supplemented as required [1 mM glutamine/ 1 mM pyruvate], pH 7.4), followed by a 45–60 min equilibration at 37 °C in a non-CO₂ incubator; OCR and ECAR were recorded using a Seahorse XF96 pro analyzer (Agilent) after standard sensor cartridge hydration/calibration, acquiring baseline measurements and then performing sequential injections of glucose (Glu, [25 mM]), oligomycin (Oligo, 1 µM), FCCP (1 µM), and rotenone/antimycin A (Rot/AA, 0,5 µM each) with three measurement cycles after each injection; rates were normalized to total protein per well by lysing cells post-run and quantifying protein with a Bradford’s assay. The ECAR/OCR ratio and the ECAR–OCR energy map was generated from post-glucose-injection measurements to estimate the glycolytic-to-oxidative balance.

### 2.3. Co-culture of memory CD4 T cells with EVs-UC-MSC

Memory CD4 T cells were isolated from PBMCs from healthy donors (HD) using the EasySep™ Human Memory CD4^+^ T Cell Enrichment Kit (Cat. #19157). 2.5x10^5^ cells/well were seeded in 96-well U-bottom plates. They were activated with Dynabeads human T-activator CD3/CD28 beads (Invitrogen), supplemented with IL-2 [250U/ml]. The EVs-UC-MSC_naive_, EVs-UC-MSC_glyco_ or EVs derived from naive UC-MSCs electroporated with miR-365a-5p were added on day 0 (1x10^8^ particles). After 4 days, the Th1, Th17 and Treg cell populations were analyzed by FACS, only in live cells according to the expression of IFN-y, IL-17 and Foxp3 respectively.

### 2.4. Co-culture of B cells with EVs-UC-MSC

B cells were isolated from PBMCs from HD by fluorescence-activated cell sorting using the BD FACSAria™ (BD Pharmingen), based on the expression of CD19 and CD20 surface markers. Sorted B cells were then co-cultured in the presence or absence of EVs-UC-MSC_naive_ or EVs-UC-MSC_glyco_. Cell viability was assessed at 24-, 42-, and 72-hours post-culture using an Annexin V/Propidium Iodide (PI) apoptosis detection assay (BD Pharmingen). 48h of culture, supernatants were collected, and interleukin-10 (IL-10) levels were quantified using an ELISA kit (R&D Systems).

### 2.5. Flow cytometry analysis (FACS)

Cell proliferation and differentiation of T cells were quantified by flow cytometry. For analysis of mouse lymph nodes and peripheral blood and human peripheral blood mononuclear cells, cells were incubated with fluorophore-conjugated specific antibodies directed against CD8, CD25, at 4°C for 20 minutes. For intracellular staining, cells were first stimulated with phorbol myristate acetate (PMA) (50ng/ml; merk, Germany) and Ionomycin (1mg/ml; Merck, Germany) in the presence of Brefeldin A (10mg/ml; Sigma , Merck, Germany) for 4 hours, after which time they were stained on their surface with antibodies against CD8, CD25 (BD Pharmingen) and labeled with LIVE/DEAD Fixable near-IR (Invitrogen, Thermo Fisher, USA) to analyze only live cells. The cells were then fixed and permeabilized with Foxp3 Cytofix/Cytoperm solution (eBioscience, USA) for 40 minutes. Finally, antibodies against CD4, IFN-y, IL17 (BD Pharmingen) and Foxp3 (eBiosciencie) were added diluted in Buffer Perm/Wash solution (eBioscience, USA) for 30 minutes.

### 2.6. Mouse model for DTH and paw swelling measurements

Mice from 8 to 12 weeks of age of the C57BL/6 strain were used. For this purpose, 1 mg/ml of ovalbumin (albumin from chicken egg white, OVA) in complete Freund's adjuvant (Sigma-Aldrich, Merck Germany) was injected intradermally into the base of the mouse tail (lower back). Five days later a booster injection of ovalbumin was performed directly into the right hind paw of the mouse concomitantly with 2x10^8^ EVs-UC-MSC_naive_ or EVs-UC-MSC_glyco_ and PBS (control) was injected into the left paw. Paw swelling was measured 24 hours after the booster and then the mice were euthanized. Blood and popliteal lymph nodes were analyzed by flow cytometry to quantify subpopulations of anti- or pro-inflammatory CD4 T lymphocytes.

### 2.7. Murine Collagen-Induced Arthritis (CIA) model

Arthritis was induced in 9-week-old DBA/1JRj mice. Bovine type II collagen (bCII, 2mg/ml) was diluted in acetic acid (0.05M) and emulsified in complete Freund's adjuvant (Thermoscientific, Rockford, IL, USA). 100µl of the emulsion was injected intradermally at the base of the mouse tail (lower back) on day 0. On day 21, 100µl of a bCII emulsion with incomplete Freund's adjuvant was administered in the same manner. On day 18 and 24, 2x10^8^ EVs-UC-MSC_naive_ or EVs-UC-MSC_glyco_ were administered by intravenous injection. The clinical signs of arthritis were graded according to the swelling of the hind paws and the signs of inflammation of the fingers of the hind paws and hands (front paws) of the mice as we previously described^13^. Clinical signs were recorded every 2 days after day 21. On the day of euthanasia, blood, lymph nodes, and spleens were collected for immune cell analysis. The hind limbs were fixed in 4% formaldehyde for histological analysis.

### 2.8. Cytokine measurements

Supernatants from *in vitro* or *ex vivo* cultures (EVs-UC-MSC_naive_ or EVs-UC-MSC_glyco_ treated cells) with PBMCs, B cells, draining lymph node (LN) or memory CD4 T cells were collected and stored at -40°C. Quantification of IL-10 levels was assessed using the ELISA kit, Human IL-10 DuoSet (R&D Systems, USA). Following the manufacturer's instructions.

### 2.9. Edge-seq data import and preprocessing

Raw miRNA count data were provided by the biotechnology company FIRALIS S.A (Huningue, France) using the HTG EdgeSeq miRNA whole transcriptome targeted sequencing assay (HTG EdgeSeq, HTG Molecular Diagnostics, Tuscon, AZ, USA). The data were delivered in a CSV file (miRNA-counts.csv) where rows correspond to miRNAs and columns to multiple replicates. These data were loaded into R as a DGEList object (via edgeR) for initial quality checks, including Q-Q plots to assess distributional characteristics. Only samples relevant to the Control versus Oligomycin comparison were retained. Specific details about microRNA transcriptomic analysis are provided in the supplementary material.

### 2.10. miRNA sequencing pipeline

Small RNA sequencing and downstream bioinformatics analyses were performed on EV-derived RNA (EV miRNA cargo) obtained from EVs-UC-MSC_glyco_ and EVs-UC-MSC_naive_, and not on RNA from treated recipient cells. Input data consisted of a miRNA count matrix (2067 detected miRNAs) with samples labeled Ctrl_1, Ctrl_3, Ctrl_4, Olig_1, Olig_3, Olig_4. One control and one oligomycin sample (suffix '_2') were excluded from downstream analyses, and all reported results correspond to this filtered dataset. Raw counts were inspected using quantile-quantile (Q-Q) plots, relative log expression (RLE) plots and principal component analysis (PCA) to evaluate global distributional differences and sample structure prior to differential expression testing. To reduce technical and other unwanted variation, counts were normalized and adjusted using RUVSeq with the RUVr procedure (k = 1), which estimates latent factors of unwanted variation from model residuals and incorporates them into normalization^14^.

### 2.11. Differential Expression Analysis

Differential expressions between oligomycin and control was assessed with edgeR (version 3.32.1) using the negative binomial model and exact test (non-paired analysis)^15^. P-values were adjusted to control the false discovery rate (FDR) using the Benjamini-Hochberg procedure^16^. Unless otherwise indicated, miRNAs were considered differentially expressed at FDR < 0.05 and |log2 fold change| > 1. A paired generalized linear model (GLM), including a pair factor derived from the sample suffix, was additionally run as a sensitivity analysis. Significant miRNAs were visualized as a clustered heatmap using log2(normalized counts + 1) and a viridis color scale. Volcano plots were generated with Enhanced Volcano *(Blighe K, Rana S, Lewis M. EnhancedVolcano: Publication-ready volcano plots with enhanced colouring and labeling. R package version. 2019 Oct 14;1(0):10-8129.)*.

### 2.12. Target annotation and functional enrichment

We focused enrichment analyses on upregulated miRNAs because these represent miRNAs enriched in EVs-UC-MSC_glyco_, which are more readily interpretable as potential mediators transferred to recipient cells (putative gain-of-function). Downregulated miRNAs were comparatively few and were not prioritized for mechanistic enrichment in this study. Putative and validated miRNA-target interactions were retrieved using multiMiR, which integrates multiple external miRNA-target databases^17^. Functional enrichment of target gene sets was performed with g:Profiler through the gprofiler2 R package (GO:BP, GO:MF, TF and Reactome sources; multiple testing correction by g:Profiler)^18^,^19^. Enrichment outputs were further summarized into presence/absence heatmaps and a ranked table of immune-related pathways across miRNAs.

### 2.13. Sankey diagram for TF–immune intersections

A Sankey diagram, generated using plotly library in Python, illustrated how enriched TFs (left) and immune terms (right) intersect through shared miRNAs. Each link’s width denotes the number of miRNAs connecting a specific TF and immune term, providing an integrative view of potential co-regulation events.

### 2.14. Artificial loading of EVs with Neon Transfection system

Extracellular vesicles (EVs) derived from non-glycolytic MSCs were loaded by electroporation using the Neon Transfection System (Thermo Fisher Scientific), following the manufacturer’s instructions. Briefly, 2 × 10⁹ vesicles were mixed with 360 nM miR-365a-5p mimic (HY-R00765) and electroporation buffer R (MPK10096T). Electroporation was performed using a pulse width 20 ms, a voltage of 850 V, and 5 pulses, as specified by the manufacturer. In addition, the same number of vesicles was electroporated with 360 nM of a miRNA mimic negative control (#4464058). All electroporated EV samples were incubated at 37 °C for 30 minutes and subsequently at 4 °C overnight to allow membrane integrity recovery. To remove unloaded microRNA remaining in the buffer, each sample was treated with RNase A (0.2 mg/mL) (Thermo Fisher Scientific) for 30 minutes at 37 °C. RNase A activity was inactivated by storing the samples at -80 °C for 24 hours. Electroporated EVs were directly used for RNA extraction and detection of miR-365a-5p by RT-qPCR. For in vitro assays aimed at evaluating miRNA transfer to target cells, electroporated EVs were washed by ultracentrifugation at 100,000 × g for 1 hour at 4 °C in 1× PBS.

### 2.15. Detection of native miR-365a-5p and miR-4478

The expression of native miRNAs in EVs was assessed by total RNA extraction using TRIzol, according to the manufacturer’s recommendations. RNA concentration was determined using a NanoDrop spectrophotometer (Thermo Scientific). cDNA was synthesized from 120 ng of RNA extracted from 2 × 10⁹ vesicles derived from non-glycolytic and glycolytic UC-MSCs using TaqMan MicroRNA Reverse Transcription Assays (Applied Biosystems) for miR-365a-5p (ID 8366141_1) and miR-4478 (ID P04012807). qPCR analysis was performed using an AriaMx Real-Time PCR System (Agilent) with TaqMan Universal Master Mix II no-UNG (Applied Biosystems, #4440040). The fold change of miR-365a-5p and miR-4478, were calculated based on cycle threshold (Ct) values.

### 2.16. Detection of miR-365a-5p in loaded EVs

The expression of miRNA loaded into EVs was evaluated by total RNA extraction using TRIzol, following the manufacturer’s recommendations. RNA concentration was determined using a NanoDrop spectrophotometer (Thermo Scientific). cDNA was synthesized from 60 ng of RNA extracted from 2 × 10⁹ vesicles derived from non-glycolytic MSCs using the TaqMan MicroRNA Reverse Transcription Assay for miR-365a-5p (Applied Biosystems, ID 8366141_1). qPCR analysis was performed using an AriaMx Real-Time PCR System (Agilent) with TaqMan Universal Master Mix II no-UNG (Applied Biosystems, #4440040). Relative miR-365a-5p fold change was calculated based on cycle threshold (Ct) values.

### 2.17. Effect of miR-365a-5p on memory CD4⁺ T cells

To evaluate the effect of miR-365a-5p on memory CD4⁺ T cells, cells were isolated from healthy donors, and 2,5 x 10^5^ cells per well were activated using anti-CD3/CD28 dynabeads human T activators (Gibco, #11132D) and treated with 1 × 10⁸ EVs loaded with miR-365a-5p for 72 hours. T-cell differentiation was subsequently quantified by flow cytometry. Th1, Th17, and Treg cell populations were analyzed by FACS exclusively within live cells, based on the expression of IFN-γ, IL-17, and Foxp3, respectively.

### 2.18. Plots and statistical analysis

Graphical representations and statistical analyses were made with GraphPad Prism to both plot graphs and statistically analyze the data. First, the distribution of the data was analyzed and those data that presented a normal distribution were analyzed by a parametric test. To compare the data between 3 groups, normality was first checked, and the one-way ANOVA test was used followed by Tukey's multiple comparison test. The p value 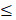
0.05 was considered significant. All FACS data were acquired using FACS Canto II (Becton Dickinson BD) and CITEK'S Aurora (Biosciences). Analysis was performed using the FLOWJO software (Becton Dickinson BD).

## 3. Results

### 3.1. Characterization of EV derived from glycolytic MSC

**Figure 1:**
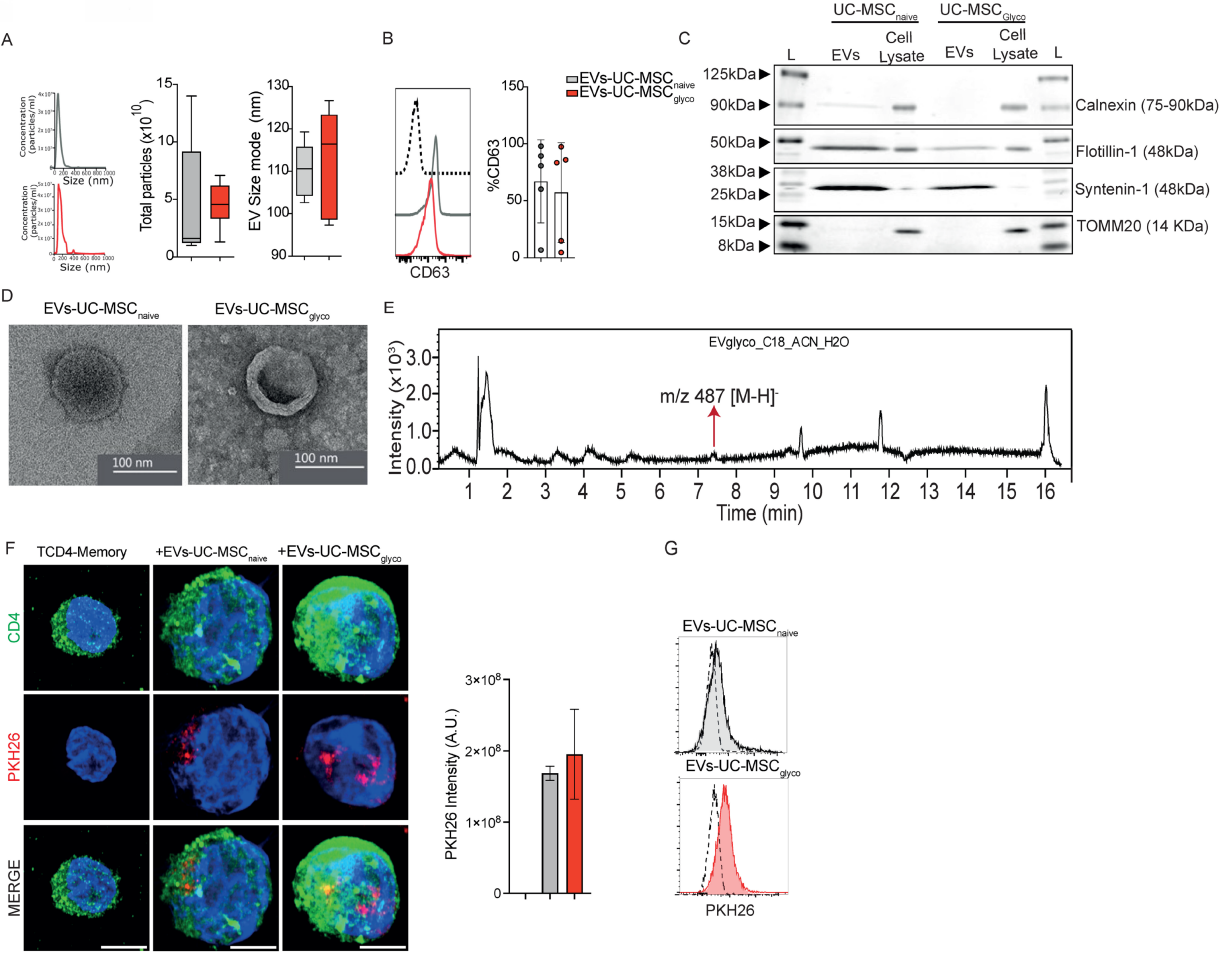
** Oligomycin does not alter the physical or molecular characteristics of extracellular vesicles (EVs) derived from UC-MSCs, and no oligomycin traces are detected in EV preparations by mass spectrometry. (A)** Nanoparticle Tracking Analysis (NTA) showing the concentration and size distribution (mode diameter) of EVs. **(B)** Flow cytometry analysis confirming the presence of classical EV surface marker CD63 in UC-MSC-derived EVs. **(C)** Western blot analysis showing the expression of EV markers syntenin and flotillin, and the absence of the cellular contaminants TOMM20 and calnexin, confirming EV purity. Bands are shown from left to right** (D)** Transmission Electron Microscopy (TEM) images of EVs from naive and glycolytic UC-MSCs (EVs-UC-MSC_naive_ and EVs-UC-MSC_glyco_), showing typical vesicle morphology and a bilayer membrane structure. **(E)** Base Peak Chromatogram (BPC) of an EV extract from oligomycin-pretreated MSCs, acquired in negative ESI mode using a C18 UHPLC column, confirming the absence of oligomycin residues. **(F)** Airyscan microscopy and Flow cytometry **(G)** analysis showing the uptake of PKH26-labeled UC-MSC-derived EVs (red) by purified memory CD4^+^ T cells (green, CD4-stained). Quantification of PKH26 fluorescence intensity following EV uptake by microscopy, expressed as arbitrary units (A.U.), indicating relative EV internalization. Red fluorescent puncta indicate internalized EVs within CD4^+^ T cells. Scale bar=5μm.

EVs were isolated by differential ultracentrifugation from naive UC-MSCs (EVs-UC-MSC_naive_) and glycolytic UC-MSCs (EVs-UC-MSC_glyco_). Nanoparticle tracking analysis (NTA) showed similar particle counts and size distributions for both (Figure 1A). Surface marker analysis confirmed CD63 expression (Figure 1B) and CD81 expression (Figure S1A) while CD9 expression was consistently low across most samples (data not shown). Western blotting revealed Flotilin 1 and Syntenin, with no Tomm20 or Calnexin contamination (Figure 1C). TEM confirmed typical lipid bilayer-enclosed vesicles (Figure 1D).

To rule out oligomycin contamination, LC-MS was performed. Control samples of oligomycin A, B, and C showed peaks at 5.4, 4.2, and 6.6 min (m/z 789, 803 and 773, respectively) (Figure S1B-D). No matching peaks were detected within EV samples, except for a minor, unrelated peak (m/z = 478) (Figure 1E), confirming the absence of residual drug. To determine the metabolic state of UC-MSCs upon activation with oligomycin, we performed a real-time metabolic assay (Seahorse) at 24, 48, and 72 hours after activation. Our results showed that the glycolytic state of UC-MSCs was maintained for up to 72 hours, without a significant decrease in glycolytic metabolism (Figure S2A-D).

### 3.2. EV derived from glycolytic MSCs exert immunosuppressive effects by IL-10 production

We next evaluated the role of EVs-UC-MSC_glyco_ on total PBMCs, as well as on purified memory CD4⁺ T cells and B cells, given their critical involvement in autoimmune diseases such as arthritis. We first evaluated the internalization of EVs and for that purpose PKH26-labeled EVs were incubated with CD4^+^ T cells. Flow cytometry and confocal microscopy confirmed uptake of both EVs types (Figure 1F-G).

We then performed a dose response analysis to determine the effective dose of EVs-UC-MSC_glyco_. For this purpose, PHA-stimulated PBMCs were isolated from healthy donors and treated with increasing doses of EVs derived from UC-MSC_naive_ or EVs-UC-MSC_glyco_ (1×10⁶, 1×10⁸, 2× 10⁸, and 5 ×10⁸ particles) (Figure S3). Our results showed that the most effective dose of EVs-UC-MSC_glyco_ was 1×10⁸ particles, whereas EVs-UC-MSC_naive_ required higher doses or showed no significant effect in inhibiting the Th1 response and promoting Treg generation in PBMCs (Figure S3). Therefore, for all subsequent experiments, we used 1 × 10⁸ particles as the effective dose of EVs-UC-MSC_glyco_. The dose of 1×10⁸ was equivalent to 0.53 ± 0.11 µg for EVs-UC-MSC_naive_ and 1.38 ± 1.19 µg for EVs-UC-MSC_glyco_ (mean ± SD, n = 4). Accordingly, a two-fold increase in EV particle number resulted in an approximately proportional increase in protein content.

Protein yield per milliliter of EV preparation was calculated and is reported as mean ± SD from four independent experiments (n = 4). The corresponding values were 411.5 ± 197.5 µg/mL for EVs-UC-MSC_naive_ and 471.5 ± 210.9 µg/mL for EVs-UC-MSC_glyco_.

**Figure 2:**
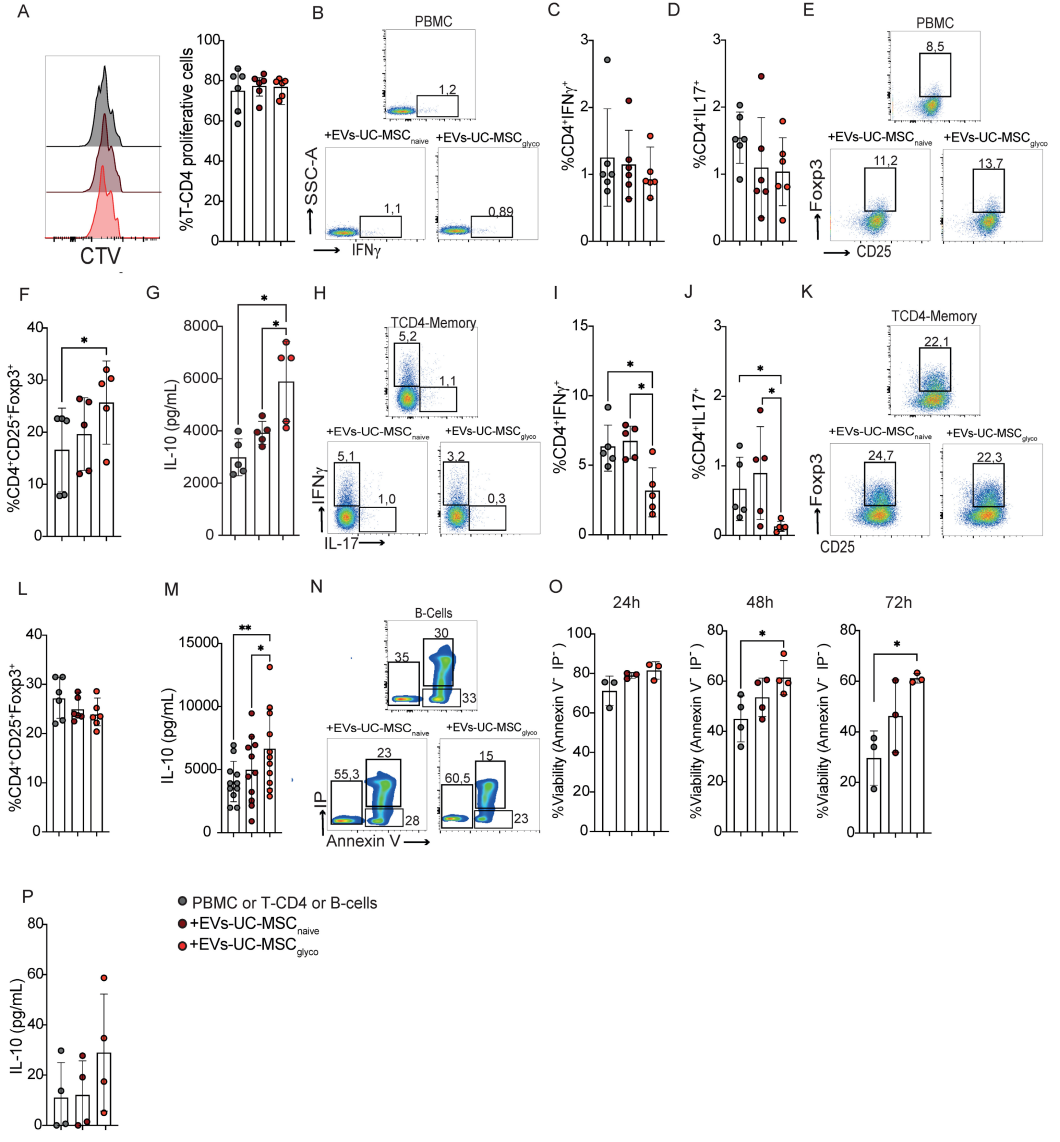
** EVs derived from glycolytic UC-MSCs exert enhanced immunomodulatory effects through increased IL-10 production.** The immunosuppressive potential of EVs-UC-MSC_naive_ and EVs-UC-MSC_glyco_ were assessed with PHA-activated human PBMC for 5 days. **(A)** Percentage of proliferating CD4^+^ T cells in PBMC cultured alone (gray), with EVs-UC-MSC_naive_ (brown) or with EVs-UC-MSC_glyco_ (red). Proliferation of CD4^+^ cells was quantified by FACS. **(B-D)** The T-cell pro-inflammatory phenotype (IFNγ and IL17 production for Th1 and Th17, respectively) were evaluated by FACS. **(E-F)** The T-cell anti-inflammatory phenotype (Treg) was evaluated by FACS. Results represent the mean ± SD of 5 independent experiments. * p < 0.05 (one-way ANOVA). **(H-J)** Representative flow cytometry dot plots **(H)** and quantification of proinflammatory Th1 (IFN-γ^+^) **(I)** and Th17 (IL-17^+^) **(J)** Memory T-CD4^+^ T cells after activation with CD3/CD28 beads, treated or not with either EVs-UC-MSC_naive_ or EVs-UC-MSC_glyco_. **(K–L)** Representative dot plot **(K)** and frequency **(L)** of CD25^+^ Foxp3^+^ regulatory T cells (Tregs) among memory CD4^+^ T cells under the same treatment conditions. Gray bars: activated memory CD4^+^ T cells alone; brown: treated with EVs-UC-MSC_naive_ red: treated with EVs-UC-MSC_glyco_. **(G-M)** IL-10 levels in culture supernatants of PBMC **(G)** and CD4^+^ T **(M)** measured by ELISA, showing increased IL-10 production following treatment with EVs-UC-MSC_glyco_. **(M–O)** Flow cytometry analysis of B cells apoptosis pretreated or not with EVs-UC-MSC_naive_ or EVs-UC-MSC_glyco_, based on Annexin V and propidium iodide (PI) staining. **(N)** Representative dot plots and **(O)** quantification of viable and late apoptotic B cells. **(P)** IL-10 levels in B cell culture supernatants after 4 days of treatment with or without EVs-UC-MSC_naive_ or EVs-UC-MSC_glyco_. Data is presented as the mean ± SD with at least 4 independent experiments. Statistical significance was assessed by one-way ANOVA or Kruskal–Walli’s test followed by Tukey’s or Dunn’s multiple comparison test. *p < 0.05, **p < 0.01.

After defining the effective dose of EVs-UC-MSC_glyco_, we evaluated their immunomodulatory effects on PHA-stimulated PBMCs, CD4⁺ memory T cells, and B-cell survival *in vitro*. Our results showed that PHA-stimulated PBMCs treated with either EVs-UC-MSC_naive_ or EVs-UC-MSC_glyco_ exhibited no changes in CD4⁺ T cell proliferation or Th1/Th17 frequencies (Figure 2A–D). However, treatment with EVs-UC-MSC_glyco_ significantly increased the frequency of CD4⁺CD25⁺ Foxp3⁺ regulatory T cells and IL-10 production (Figure 2E–G), suggesting selective tolerogenic immunomodulatory activity.

In purified memory CD4⁺ T cells, EVs-UC-MSC_glyco_ significantly reduced the frequencies of IFN-γ⁺ and IL-17⁺ cells without altering Treg levels (Figure 2H–L). However, consistent with the PBMC results, EVs-UC-MSC_glyco_ also significantly enhanced IL-10 production in memory CD4⁺ T cells (Figure 2M), indicating their ability to reduce inflammation while promoting a more tolerogenic cytokine environment via IL-10. To further investigate the role of IL-10 in the immunomodulatory effects of EVs-UC-MSC_glyco_, we performed inhibition assays using blocking antibodies against IL-10 and its receptor. Blockade of either target significantly reduced IL-10 production, with a stronger effect observed upon IL-10 neutralization. Notably, reversal of the suppressive effects of EVs-UC-MSC_glyco_ was more pronounced upon IL-10 receptor blockade, supporting a key role for IL-10 signaling in mediating the suppression of pro-inflammatory CD4⁺ T-cell responses (Figure S4).

Finally, we assessed the impact of EVs-UC-MSC_glyco_ on CD19⁺CD20⁺ B cells, which are known to be highly sensitive to changes in their microenvironment, and whose survival and tolerogenic functions are critical in autoimmune contexts. Our results showed that EVs-UC-MSC_glyco_ enhanced both B cell viability (Figure 2N–O) and IL-10 production (Figure 2P).

Altogether, these findings demonstrate that EVs-UC-MSC_glyco_ modulate effector memory CD4⁺ T cells and promote IL-10 production in both T and B cells, supporting their potential as tolerogenic immunomodulators in autoimmune diseases such as RA.

### 3.3. Attenuation of DTH by EVs derived from naive or glycolytic UC-MSCs through Tregs induction and IL-10 production

**Figure 3:**
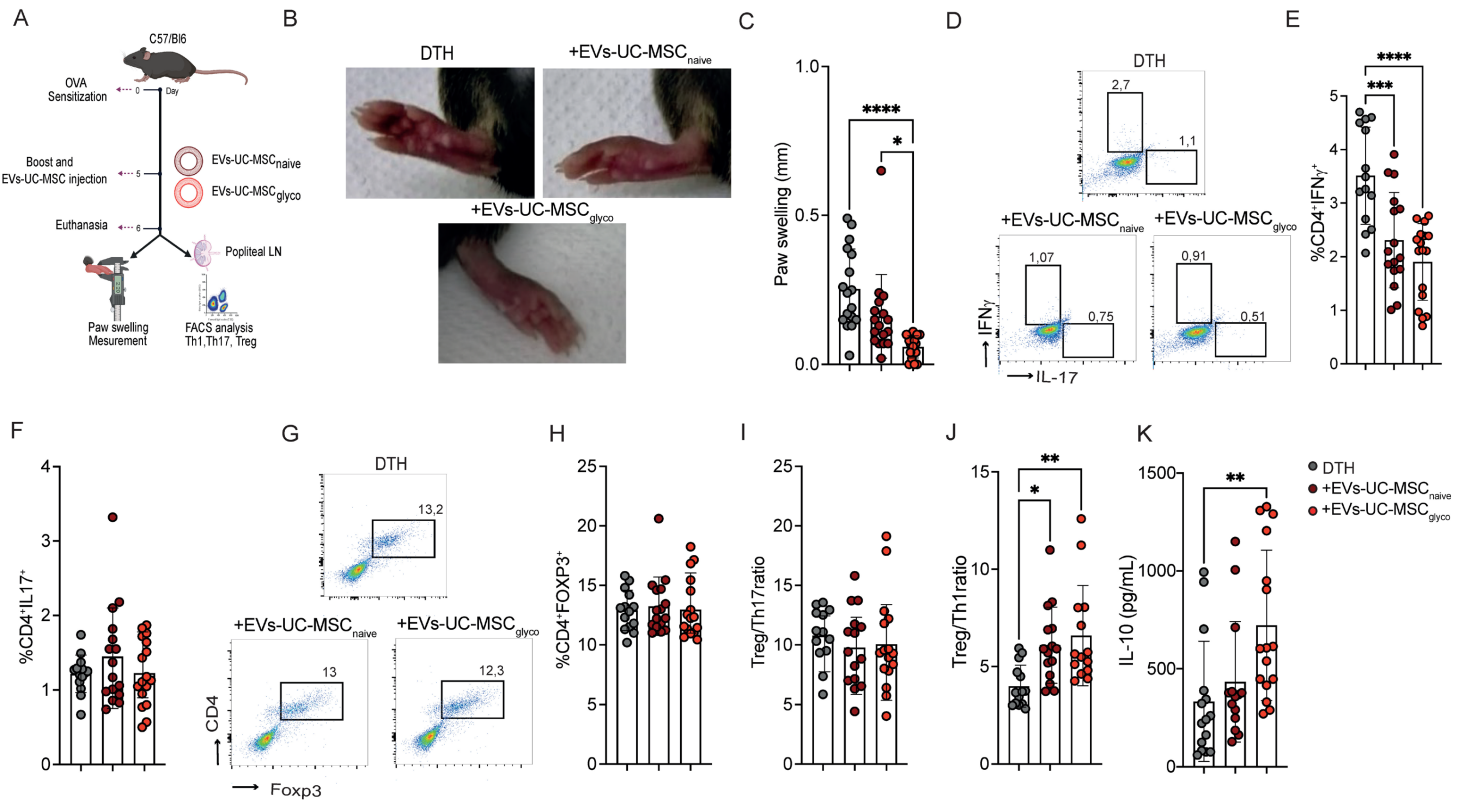
** Glycolytic MSC derived EVs show enhanced therapeutic effect in a murine DTH model. (A)** Experimental design of the DTH murine model. **(B-C)** Paw swelling measurement 24 h after antigen boost and injection of EVs-UC-MSC_naive_ or EVs-UC-MSC_glyco_. **(D–H)** After euthanasia, proinflammatory Th1 **(D, E)** and Th17 **(D, F)** lymphocytes and anti-inflammatory Treg cells **(G, H)** present in the popliteal lymph nodes of DTH mice (gray) or DTH mice treated with EVs-UC-MSC_naive_ (brown) or EVs-UC-MSC_glyco_ (red) were measured by FACS. Treg/Th17 ratio **(I)** Treg/Th1 ratio **(J)** was also calculated. **(K)** IL-10 levels in LN supernatants of non-treated (grey bars) or treated with EVs-UC-MSC_naive_ (brown bars) or EVs-UC-MSC_glyco_ (red bars) for 24h after euthanasia. Data represents SD from three independent experiments with at least 14 animals per experimental group; *p < 0.05, **p < 0.01, ***p < 0.001, ****p < 0.0001 (one-way ANOVA followed by Tukey’s multiple comparison test). Unless noted otherwise, comparisons are made against untreated DTH mice.

In the DTH model (a well-established approach to assess cell-mediated immune responses driven by Th1 and Th17 activity) (Schematic representation of the model in Figure 3A), EVs-UC-MSC_glyco_ reduced paw swelling more effectively than control EVs (Figure 3B-3C). Both EV treatments led to a reduction of CD4⁺IFNγ⁺ (Th1) cells (Figure 3D and E) in the LNs, while CD4⁺IL-17⁺ (Th17) cell frequencies remained unchanged (Figure 3D and F). Although the overall frequency of Tregs was not significantly altered (Figure 3H), both EVs promoted an increase in the Treg: Th1 ratio (Figure 3J), indicating a shift toward a more regulatory immune profile. Notably, EVs-UC-MSC_glyco_ treatment was associated with a more pronounced effect, including a significant increase in IL-10 production in culture supernatants (Figure 3K), further supporting their enhanced immunosuppressive capacity.

### 3.4. EVs-UC-MSC_glyco_ reduce CIA incidence and severity through the induction of IL-10-producing B cells

**Figure 4:**
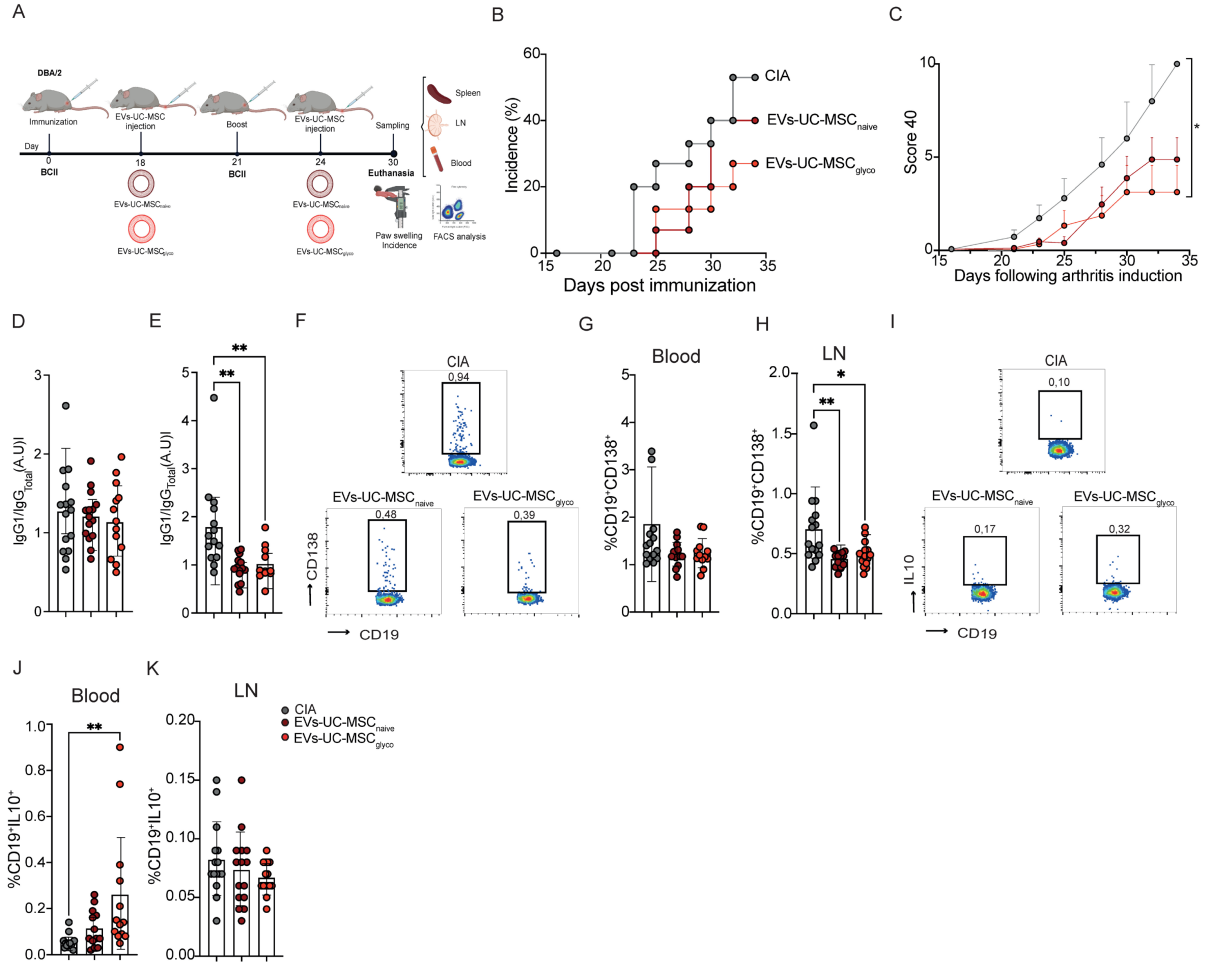
** EVs-UC-MSC_glyco_ treatment prevents inflammatory responses and joint damage of CIA mice via IL 10^+^ B cells. (A)** Experimental design of the CIA murine model. **(B)** Incidence of mice with inflammation in the collagen-induced arthritis (CIA) model until day 35 at euthanasia (n=15 biological replicates). **(C)** Inhibition of inflammation as measured by the global clinical score 40 in the same mice as in (B). **(E-F)** Type II collagen-specific IgG1/IgG_total_ antibody ratios were quantified in sera at day 16 **(E)** and at day 22 **(F)** of CIA mice (gray) or mice treated with EVs-UC-MSC_gnaive_ (brown) or EVs-UC-MSC_glyco_ (red) during CIA by a modified ELISA assay. **(G-K)** Frequency of CD19^+^CD138^+^ plasma cells in the blood **(H)** lymph nodes **(I)** and CD19+IL10+ Breg cells in blood **(J)** and in lymph nodes **(K)** from CIA mice (gray), mice treated with EVs-UC-MSC_naive_ (brown) or EVs-UC-MSC_glyco_ (red). Data were analyzed by one-way ANOVA with Tukey’s multiple comparison test, with at least n=12 biological replicates and *p < 0.05, **p < 0.01, ***p < 0.001, ****p < 0.0001.

In CIA mice, EVs were administered intravenously on days 18 and 24 post-immunization (Figure 4A), based on previously established efficacy at these time points^13^. Since this model involves intravenous administration of EVs, we first assessed EV biodistribution 6h post-injection in mice. EVs predominantly accumulated in the lungs, followed by distribution to peripheral organs, including the spleen, indicating that EVs can reach tissues relevant to CIA pathogenesis in this autoimmune setting (Figure S5).

EVs-UC-MSC_glyco_ showed a significant reduction in disease incidence compared to untreated arthritic mice (Figure 4B). Also, these animals exhibited a significantly lower global arthritis clinical score (Figure 4C), lower fore paws score (Figure S6A), paw swelling (Figure S6B) and hind paw (Figure S6C). In contrast, treatment with EVs-UC-MSC_naive_ did not significantly reduce the overall clinical score. However, a downward trend in both disease incidence and severity was observed, along with a decrease in inflammation in the fore paws compared to untreated mice (Figure S6A). Histological analysis further confirmed the therapeutic benefit of EVs-UC-MSC_glyco_, showing a marked reduction in immune cell infiltration in the joints of treated mice compared to untreated controls (Figure S6D).

We then measure the ratio of collagen type II-specific IgG1 to total IgG antibodies in sera at days 16 (Figure 4D) and 22 (Figure 4E), finding a significant reduction of the IgG1/IgG_total_ ratio at day 22, suggesting attenuation of the autoimmune antibody response (Figure 4E). Analysis of immune cell populations revealed no differences in CD19^+^CD138^+^ plasma cells in the blood (Figure 4F-G and 4H). Conversely, a significant reduction in this population was observed in the LN of EV-treated mice (Figure 4H). Notably, EVs-UC-MSC_glyco_ significantly increased the percentage of circulating CD19^+^IL-10^+^ regulatory B cells (Breg-like cells) in blood compared to untreated CIA mice (Figure 4J). This increase was not observed in mice treated with EVs-UC-MSC_naive_, nor in the LN of untreated mice (Figure 4K). Together, these findings supported that EVs-UC-MSC_glyco_ enhance immunomodulatory effect relative to EVs-UC-MSC_naive_ in the CIA model, and are associated with increased circulating IL-10-producing regulatory B cells (Breg).

### 3.5. Enhanced attenuation of CIA by EVs derived from glycolytic MSCs is associated with decreased Th1 cells and increased regulatory T cells

**Figure 5:**
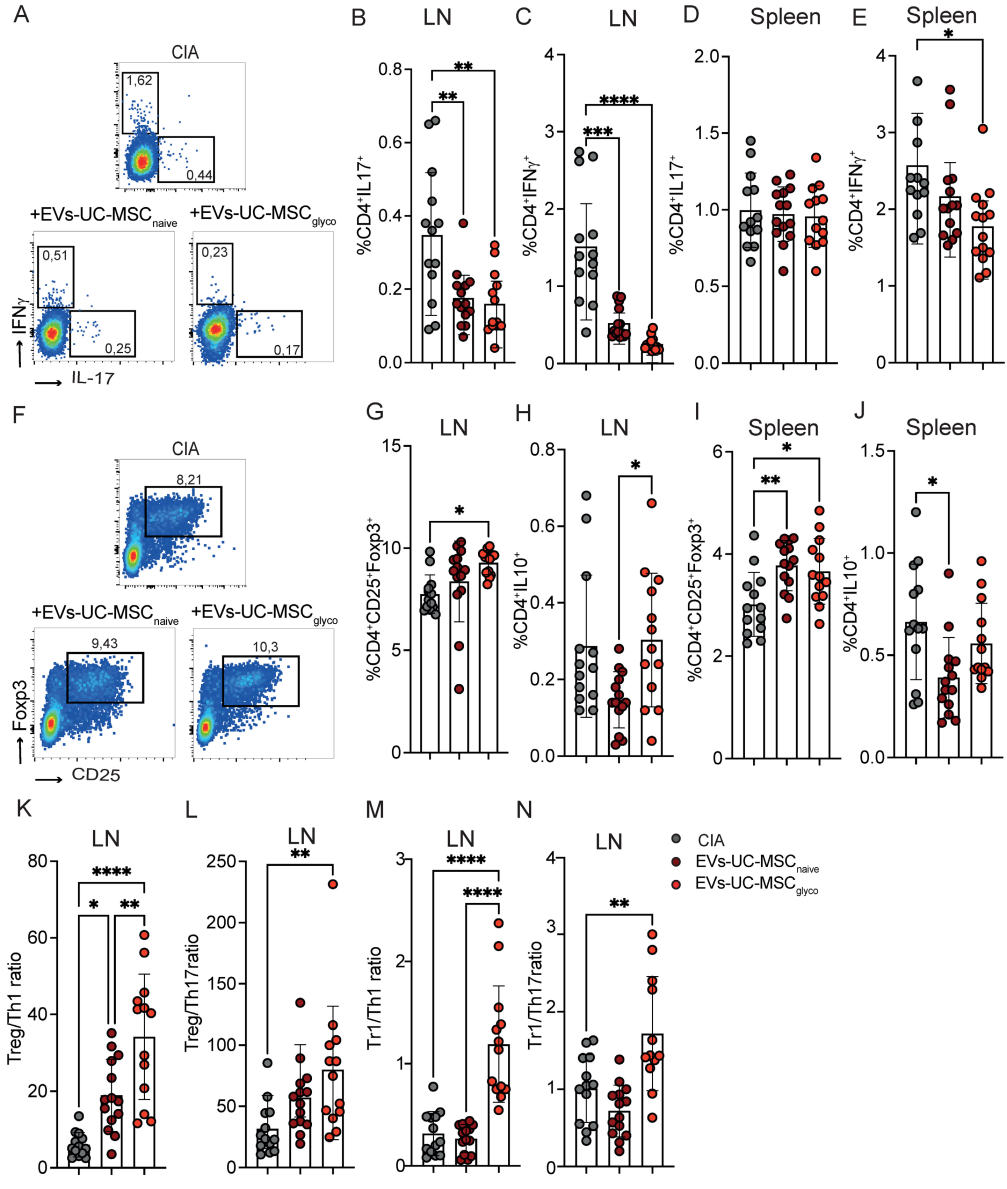
** EVs-UC-MSC_glyco_ modulate T lymphocyte populations in CIA mice. (A–J)** Flow cytometry analysis of T cell subsets in collagen-induced arthritis (CIA) mice (gray), and mice treated with EVs-UC-MSC_naive_ (brown) or EVs-UC-MSC_glyco_ (red). **(B, D)** Frequency of proinflammatory CD4^+^IL-17^+^ Th17 cells in the lymph nodes **(B)** and spleen **(D)**. **(C, E)** Frequency of CD4^+^IFN-γ^+^ Th1 cells in the lymph nodes **(C)** and spleen **(E)**.** (G, I)** frequency of CD4^+^CD25^+^ Foxp3^+^ regulatory T cells (Tregs) in the lymph nodes **(G)** and spleen **(I)**. **(H, J)** Frequency of CD4^+^IL-10^+^ type 1 regulatory T cells (Tr1) in the lymph nodes **(H)** and spleen **(J)**. **(K–N)** Immunoregulatory ratios calculated in lymph nodes: Treg/Th1 (K), Treg/Th17 (L), Tr1/Th1 **(M),** and Tr1/Th17 **(N)**, highlighting the shift in T cell balance toward anti-inflammatory phenotypes upon EV treatment. One-way ANOVA with Tukey’s multiple comparison test was used for statistical analysis, with at least n=12 biological replicates and *p < 0.05, **p < 0.01, ***p < 0.001, ****p < 0.0001.

To further investigate the immunomodulatory mechanisms underlying EV-mediated effects in CIA, we next analyzed the frequency of key T cell subsets involved in the pathogenesis of arthritis. Both EVs-UC-MSC_naive_ and EVs-UC-MSC_glyco_ significantly reduced the percentage of pro-inflammatory Th1 and Th17 cells in the LN of treated mice compared to arthritic controls (Figure 5A-C). Notably, only mice treated with EVs-UC-MSC_glyco_ exhibited a significant reduction in splenic Th1 cells (Figure 5E) while neither treatment significantly altered the proportion of Th17 cells (Figure 5D). Moreover, treatment with EVs-UC-MSC_glyco_ induced a marked increase in CD4^+^CD25^+^Foxp3^+^ Treg cells in both the LN and spleen compared to untreated arthritic mice (Figure 5F, 5G, 5I), whereas EVs-UC-MSC_naive_ had a less pronounced effect. Interestingly, EVs-UC-MSC_naive_ treatment resulted in a reduction of CD4^+^ cells producing IL-10 in both the LNs and spleen, while this was not observed in mice receiving EVs-UC-MSC_glyco_ (Figure 5H, 5J). These findings were confirmed by the analysis of absolute cell numbers, yielding similar results (Figure S7, in absolute number), suggesting differential modulation of regulatory cytokine production.

Altogether, these findings indicate that EVs-UC-MSC_glyco_ elicit a more pronounced immunomodulatory effect than EVs-UC-MSC_naive_ in the CIA model, characterized by a selective reduction in Th1 responses and robust expansion of IL-10-producing Treg populations, indicative of a shift toward immune tolerance.

### 3.7. Differential miRNA content in EVs derived from UC-MSC_glyco_ and UC-MSC_naive_

To investigate the molecular mechanisms underlying the enhanced immunomodulatory function of EVs-UC-MSC_glyco_, we profiled their miRNA content and compared it to that from EVs-UC-MSC_naive_. To correct for technical variation and batch effects, we applied the Removal of Unwanted Variation (RUVSeq) method. After RUVr adjustment, Relative Log Expression (RLE) distributions were largely centered around zero, indicating improved between-sample comparability. Principal Component Analysis (PCA) showed that the dominant axis of variation (PC1=76.33%) captured treatment-associated differences, with oligomycin samples generally shifted relative to controls, although one oligomycin replicate remained closer to the control cluster (Figure S8).

**Figure 6:**
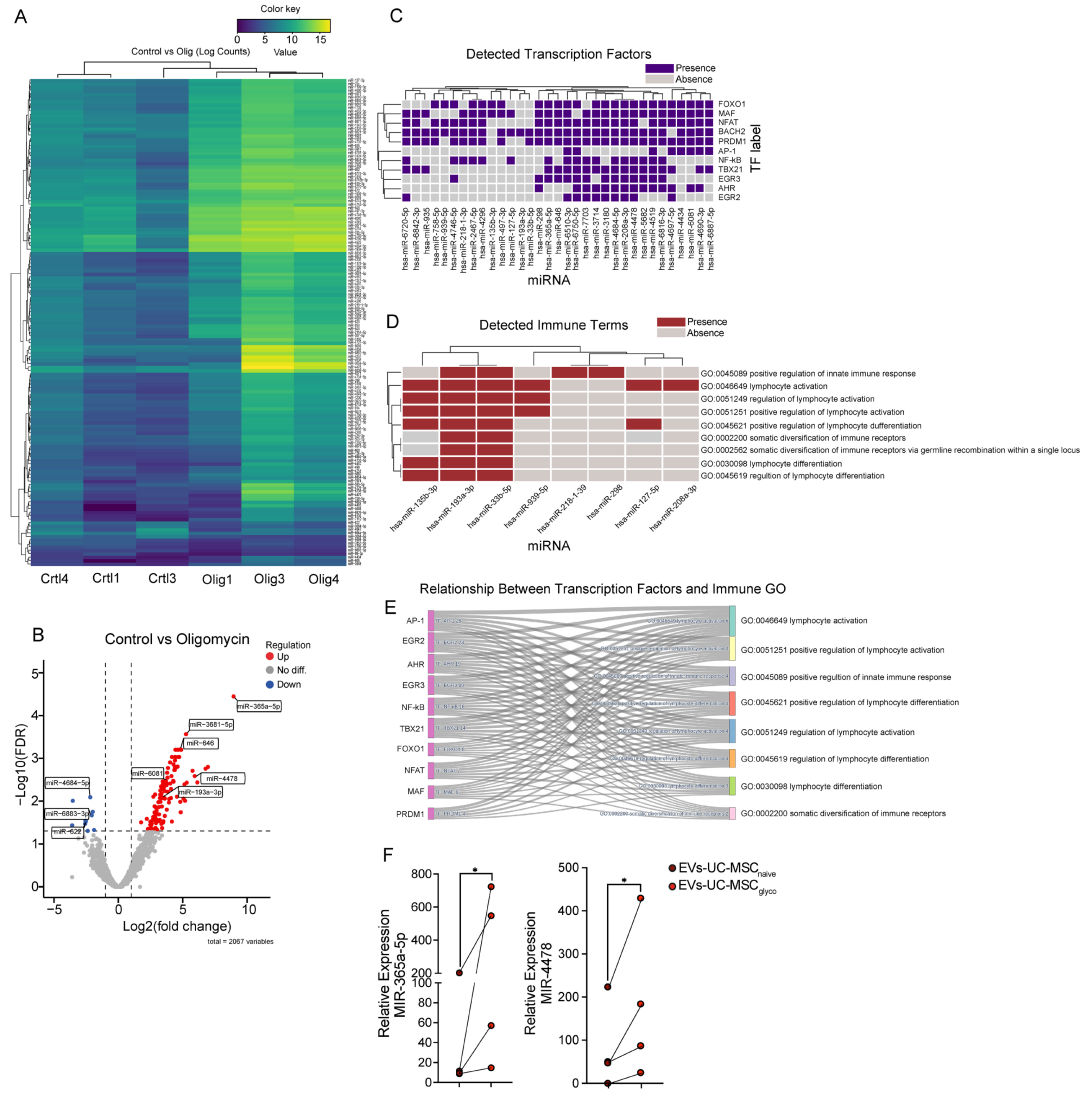
** Multi-level analysis of differentially expressed miRNAs in EV upon oligomycin treatment. (A)** Heatmap of differentially expressed miRNAs (DEMs) between Control and Oligomycin-treated samples. miRNAs were filtered by the selected differential expression thresholds, and normalized expression values [log2(normalized counts + 1)] were hierarchically clustered across miRNAs (rows) and samples (columns). The color scale indicates relative expression levels, with yellow representing higher expression and blue representing lower expression. **(B)** Volcano plot illustrates changes in miRNA expression between Control and Oligomycin conditions. The x-axis shows the log2 fold change, and the y-axis shows the -log10(FDR). Red points indicate significantly upregulated miRNAs, and blue points indicate significantly downregulated miRNAs, based on the chosen thresholds (|log2FC| > 1.0 and FDR < 0.05). **(C)** Presence/absence heatmap of enriched transcription factors (TFs) identified from the target genes of different miRNAs. Each cell indicates whether a given miRNA (columns) is associated with enrichment for a particular TF (rows). **(D)** Presence/absence heatmap of enriched immune-related GO terms associated with different miRNAs. Terms were selected based on a curated immune-related keyword list. Each cell indicates whether a given miRNA (columns) is linked to a particular immune GO term (rows). **(E)** The Sankey diagram illustrates the overlap between TFs (left) and immune-related GO terms (right) for all miRNAs. The width of each link corresponds to the number of miRNAs jointly targeting TF and an immune term, highlighting the interplay between transcriptional regulation and immune-related pathways. **(F)** qPCR validation of miRNA 365a-5p and miRNA 4478 in EVs derived from UC-MSC_naive_ and UC-MSC_glyco**.**_Data are presented as mean ± SD from three independent UC-MSC donors (n = 3), with paired samples connected by lines. Statistical significance was assessed using a two-tailed Wilcoxon matched-pairs signed-rank test; *p < 0.05.

Across 2,067 tested miRNAs, the non-paired edgeR analysis identified 141 differentially expressed miRNAs at FDR < 0.05 and |log2FC| > 1.0 (up in oligomycin: 131; down: 10). The paired GLM sensitivity analysis yielded 188 differentially expressed miRNAs (up: 162; down: 26). Most signals were shared between approaches (overlap: 137 miRNAs), with strong concordance of effect sizes (Pearson r = 0.96 for overlapping differentially expressed miRNAs) (Figure 6A). The volcano plot showed a strong asymmetry toward positive log2 fold changes, reflecting the predominance of upregulated miRNAs in the oligomycin condition (Figure 6B). Among the strongest induced miRNAs were miR-365a-5p (log2FC 8.95, FDR 3.57e-05), miR-3681-5p (log2FC 5.26, FDR 2.72e-04), miR-4478 (log2FC 5.92, FDR 0.003), miR-6081 (log2FC 3.70, FDR 0.003), miR-193a-3p (log2FC 3.45, FDR 0.008). Among the few repressed miRNAs were miR-3064-5p (log2FC -3.54, FDR 0.010), miR-4684-5p (log2FC -2.18, FDR 0.008), miR-622 (log2FC -2.52, FDR 0.030).

Using multiMiR, 24,643 miRNA-target records were retrieved across 76 miRNAs, representing 10,233 unique target genes. Enrichment analyses of these target sets highlighted cell cycle and cytoskeletal processes (e.g., cytokinesis-related terms) and multiple immune-associated signatures, including antigen processing and MHC class I presentation, regulation of immune system processes, leukocyte/lymphocyte activation and differentiation, and cytokine-related pathways. A heatmap (Figure 6C) illustrates the presence/absence of transcription factors (TFs) predicted to be targeted by the upregulated miRNA. This analysis identified specific miRNAs—such as miR-7703, miR-4684-5p, miR-3714, and miR-4478—that are significantly associated with key TFs involved in immune regulation, including NFAT, NF-κB, and MAF. Furthermore, an immune-specific heatmap (Figure 6D) shows the alignment of several miRNAs (notably miR-193-5p and miR-33b-5p) with predefined immune-related GO terms, such as “positive regulation of innate immune response” (GO:0045089), “lymphocyte activation” (GO:0046649), “regulation of lymphocyte activation” (GO:0051249), and “positive regulation of lymphocyte activation” (GO:0051251). A ranked summary of enriched immune-related pathways from GO:BP and Reactome is provided in Table S1.

Finally, a Sankey diagram (Figure 6E) integrates these results by linking TFs to specific immune-related GO categories, providing a visual summary of the regulatory networks potentially influenced by these upregulated miRNAs. To validate the sequencing results, we performed RT–qPCR on two representative upregulated miRNAs (miR-365a-5p and miR-4478). Consistent with the differential expression analysis, both miRNAs showed significantly higher relative expression in EVs-UC-MSC_glyco_ compared with EVs-UC-MSC_naive_ (Figure 6F), with lines connecting donor-matched preparations.

Altogether, these findings suggest that oligomycin-induced glycolysis in MSCs modifies EV miRNA cargo in a manner consistent with enhanced immunomodulatory activity and predicted miRNA-TF-immune pathways interactions.

### 3.8. EV-associated miR-365a-5p contributes to the suppression of memory CD4⁺ T-cell responses

miR-365a-5p was among the most strongly enriched and abundant EV miRNAs in UC-MSC_glyco_. Consistent with its prioritization, our miRNA target-based transcription factor (TF) analysis identified MAF (c-Maf) as a prominent TF node associated with the miR-365a-5p target network (Figure 6C). Given that IL-10–associated regulatory programs in CD4⁺ T cells are governed by coordinated transcriptional regulation of IL-10^20^ and related immunoregulatory genes, where c-Maf is a key regulator^21^,^22^, we tested whether enriching EVs with miR-365a-5p is sufficient to reproduce key IL-10/Treg-skewing effects.

**Figure 7:**
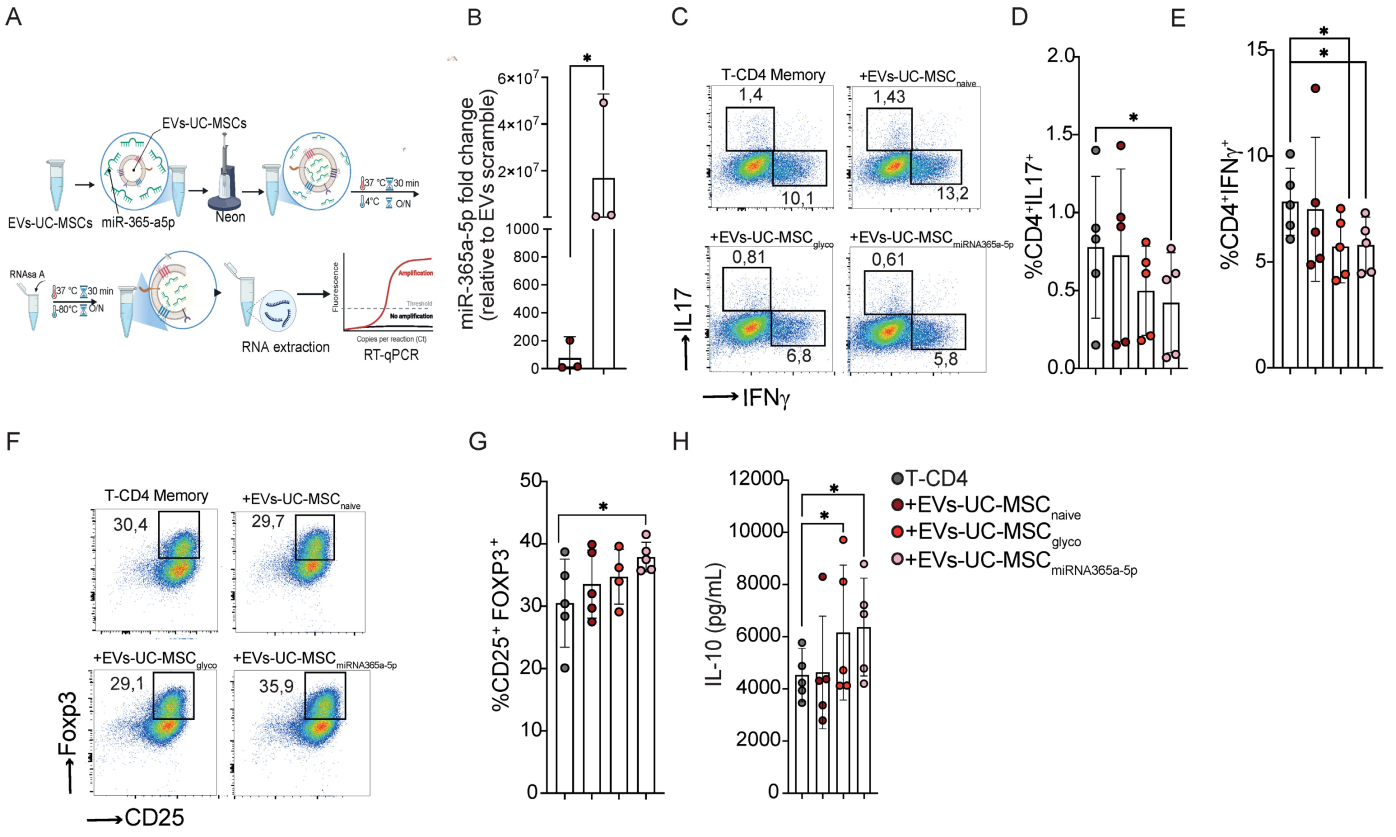
** EVs -UC-MSC induce immunoregulatory CD4⁺ memory T-cell responses through miR-365a-5p transfer. (A)** Schematic overview of the experimental workflow used to evaluate EV-charge with miR-365a-5p. **(B)** Quantification of miR-365a-5p levels in EVs-UC-MSC expressed as fold change relative to EV-UC-MSC-scramble control. **(C-E)** Representative flow cytometry dot plots **(C)** and quantification of proinflammatory Th17 (IL-17^+^) **(D)** and Th1 (IFN-γ^+^) **(E)** Memory T-CD4^+^ T cells after activation with CD3/CD28 beads, treated or not with either EVs-UC-MSC_naive_ or EVs-UC-MSC_glyco_ or EVs-UC-MSC_miRNA365a-5p_ **(F-H)** Representative dot plot **(F)** and frequency **(G)** of CD25^+^ Foxp3^+^ regulatory T cells (Tregs) among memory CD4^+^ T cells under the same treatment conditions. Gray symbols: activated memory CD4^+^ T cells alone; brown: treated with EVs-UC-MSC_naive_ red: treated with EVs-UC-MSC_glyco,_ pink: treated with EVs-UC-MSC_miRNA365a-5p._ **(H)** IL-10 levels in culture supernatants of memory CD4^+^ T under the same treatment conditions measured by ELISA. Data are presented as mean ± SD from n = 5 independent experiments using cells from different donors. Statistical analyses were performed using one-way ANOVA followed by Tukey’s multiple-comparison test. *p < 0.05.

To this end, we established a protocol using the Neon transfection system to load miR-365a-5p into EVs derived from naive UC-MSCs, resulting in a significant enrichment of this miRNA (Figure 7A). Based on the RT-qPCR readout in Figure 7B, miR-365a-5p abundance increased ~10² (control EVs) to ~10⁷ relative units in loaded EVs (on the order of ~1×10^5^-fold). Functionally, miR-365a-5p–enriched EVs significantly reduced Th17, Th1 differentiation (Figure 7C, D and E) and enhanced Treg generation (Figure 7F and G), concomitant with increased IL-10 production (Figure 7H).

Notably, these immunomodulatory effects were not directly proportional to the degree of miRNA enrichment, suggesting that additional EV-associated factors-potentially other miRNAs or components of the EV cargo-contribute to the observed suppressive phenotype and warrant further investigation.

## 4. Discussion

By comparing EVs derived from control and glycolytically reprogrammed UC-MSCs, we demonstrated that induction of glycolysis enhances the immunoregulatory properties of MSC-derived EVs both *in vitro* and *in vivo*, in two murine models of arthritis. Glycolytic EVs more effectively suppressed Th1 and Th17 responses and promoted IL-10 production, resulting in a significant reduction in delayed-type hypersensitivity (DTH) inflammation and collagen-induced arthritis (CIA) progression. These findings are consistent with previous studies showing enhanced EV function following MSC priming with IFN-γ or hypoxia, two strategies that significantly increase the glycolytic activity of MSC^6^,^23^.

Importantly, LC-MS analysis confirmed the absence of oligomycin in EV preparations, indicating that the observed immunomodulatory effects stem from MSC metabolic reprogramming rather than direct drug transfer. Based on this, we explored differences in miRNA cargo between EVs from oligomycin-treated and naïve UC-MSCs. The results indicate that glycolytic reprogramming is associated with a distinct EV miRNA profile, and our bioinformatic analyses suggest that these miRNAs could contribute to immunoregulatory programs through putative miRNA–transcription factor networks regulating key immune pathways.

While numerous clinical trials support the therapeutic potential of MSC-based interventions^24^, challenges remain, such as environmental sensitivity and limited persistence *in vivo*. EVs offer a promising, cell-free alternative that retains MSC immunoregulatory effects while mitigating concerns related to tumorigenicity, cell viability, or post-administration engraftment^25^,^26^. EVs are scalable^27^,^28^, compatible with sterile filtration^29^, stable during freeze–thaw cycles^30^ and they lack nuclear DNA or replicative potential^25^,^26^. Although ultracentrifugation may co-isolate protein contaminants, it remains a widely accepted method. To minimize immunogenicity, we replaced FBS with human platelet lysate and used serum- and antibiotic-free medium during EV collection. Notably, oligomycin treatment did not affect EV yield, and both EV populations preserved classical morphology and expression of tetraspanin markers.

Consistent with prior evidence that MSC-derived EVs mediate intercellular communication via bioactive cargo—such as cytokines, lipids, metabolites, and miRNAs—we confirmed the active uptake of UC-MSC-EVs by memory CD4⁺ T cells by confocal microscopy and FACS^31^-^36^.

Previous studies have shown that MSC-EVs can modulate both CD4⁺ and CD8⁺ T cell responses^37^-^39^. We further demonstrated that EVs-UC-MSC_glyco_ modulate immune responses more effectively than their naïve counterparts. While neither EV type significantly altered CD4⁺ T cell proliferation nor Th1/Th17 frequencies in PHA-activated PBMCs, EVs-UC-MSC_glyco_ increased CD4⁺CD25⁺Foxp3⁺ Treg generation and IL-10 production, indicating that glycolytic EVs exert immunoregulatory activity in this mixed-cell system primarily by enhancing regulatory outputs. In memory CD4⁺ T cells, only glycolytic EVs significantly suppressed Th1 and Th17 populations and enhanced IL-10 levels without affecting classical Treg frequencies, indicating a selective and targeted anti-inflammatory effect within an effector-prone subset. Moreover, EVs-UC-MSC_glyco_ improved B cell viability and induced IL-10 secretion, demonstrating a broader immunomodulatory effect that includes both T and B cell compartments.

An important assay-dependent difference emerged between mixed PBMC cultures and purified memory CD4⁺ T cells. In PHA-stimulated PBMCs, EVs-UC-MSC_glyco_ increased IL-10 and Foxp3⁺ Tregs without measurable changes in Th1/Th17 frequencies. This may reflect the strong, polyclonal nature of PHA stimulation and the complexity of PBMC cultures, where effector frequencies can be buffered by heterogeneity in responding subsets and by accessory-cell–driven inflammatory cues. In contrast, in purified memory CD4⁺ T cells activated through CD3/CD28, EVs-UC-MSC_glyco_ reduced IFN-γ⁺ and IL-17⁺ frequencies while increasing IL-10, consistent with an effect that is more readily detectable in a purified system with reduced confounding by accessory-cell signals. Notably, IL-10/IL-10R blockade partially reversed these effects, supporting a functional contribution of IL-10 signaling in the memory CD4⁺ T-cell context. Together, these findings suggest that glycolytic EVs can promote IL-10-associated regulatory programs even when shifts in Th1/Th17 frequencies are not readily detectable in highly activated mixed PBMC systems, and they underscore the value of complementary assays to capture subset-specific EV activity.

Crosstalk between IL-10-producing B and T cells may further contribute to the in vivo phenotype. IL-10-producing regulatory B cells have been shown to restrain inflammatory arthritis and to limit pathogenic effector T-cell responses, including Th17, in CIA and related models^40^,^41^. In addition to acting directly on effector T cells, Bregs can support the establishment of regulatory milieus through IL-10 and other suppressive mediators, thereby promoting regulatory T-cell programs^42^. Conversely, IL-10-producing T-cell states may help reinforce B-cell regulatory functions, suggesting a bidirectional amplification loop rather than two independent phenotypes. In this context, c-Maf represents a shared transcriptional node linked to IL-10 programs across lymphocyte lineages, including CD4⁺ T cells and IL-10-producing B cells, providing a plausible mechanistic convergence point^43^. Although our study was not designed to formally dissect T-B causality in vivo, these observations support a model in which EVs-UC-MSCglyco can engage both compartments and promote a coordinated IL-10-dominant regulatory network; future studies using co-culture dependency assays, targeted depletion, or adoptive transfer will be required to define directionality and hierarchy.

These findings expand upon prior work, such as that of Cosenza et al., where IFN-γ-primed MSC-EVs suppressed activated splenocytes, reduced IFN-γ⁺ CD8⁺ T cells, improved B cell viability, and increased Treg frequencies^13^. While naïve MSC-EVs are known to reduce Th1 responses and enhance IL-10 production^44^,^45^, our results suggest that metabolic reprogramming further augments their immunosuppressive potency and consistency.

Our *in vivo* data reinforces this observation. In the DTH model, EVs-UC-MSC_glyco_ significantly increased the Treg: Th1 ratio and IL-10 production, indicative of a favorable immunoregulatory milieu. In the CIA model, treatment with EVs-UC-MSC_glyco_ reduced disease incidence, clinical severity, and joint damage more effectively than both untreated controls and mice treated with naïve EVs. This beneficial effect was associated with significant increases in Treg/Th1, Treg/Th17, Tr1/Th1, and Tr1/Th17 ratios in lymph nodes, suggesting a systemic anti-inflammatory response driven by glycolytic reprogramming. An important limitation of our *in vivo* experiments is that they primarily represent acute challenge and early-interventions settings rather that treatment of established disease. Therefore, while our results demonstrate that EVs-UC-MSC_glyco_ can reduce inflammatory responses and attenuate CIA progression, they do not directly address whether these EVs can reverse established arthritis, which is more clinically relevant. Future studies should test therapeutic regimens in which EVs are administered after disease onset to determine their ability to treat ongoing inflammation and joint pathology.

Th1 and Th17 cells are key mediators of autoimmune pathology in CIA and other inflammatory diseases, mainly through secretion of IFN-γ and IL-17, which promote synovial inflammation and joint destruction^1^,^46^,^47^. Conversely, regulatory T cells (Tregs) and type 1 regulatory T cells (Tr1, CD4⁺IL-10⁺) are crucial for maintaining immune tolerance and suppressing pathogenic effector responses. Their restoration is considered a hallmark of effective immunotherapy in autoimmune conditions^48^. The selective increase in these regulatory-to-effector T cell ratios in response to EVs-UC-MSC_glycol_, but not to naïve EVs, highlights the added value of metabolic reprogramming in enhancing the immunosuppressive function of MSC-derived EVs. Supporting this, EVs-UC-MSC_glyco_ significantly increased the frequency of IL-10–producing regulatory B cells (CD19⁺IL-10⁺), particularly in peripheral blood, suggesting that B cell–mediated immunosuppression may also contribute to CIA attenuation.

Mechanistically, metabolic cues may alter the molecular composition of EV cargo, including miRNAs, proteins, lipids and metabolites, which in turn could shape their interactions with immune recipient cells. While our study provides miRNA profiling and in silico target/enrichment analyses, additional multiomics and functional experiments are required to delineate the relative contribution of each cargo component. To investigate this, we characterized the miRNA content of EVs and identified a distinct signature in those derived from glycolytic UC-MSCs. This profile was associated with the suppression of proinflammatory T cell pathways and the upregulation of IL-10–mediated regulatory programs. Going further in the microRNA cargo analysis, our bioinformatic analysis identified immune-relevant transcription factors (TFs) as putative nodes within the target networks of the most abundant miRNAs enriched in EVs-UC-MSC_glyco_. These included c-Maf, NFAT, NF-κB, Tbx21 (T-bet), Ahr, Foxo1, AP-1, Bach2, Egr2/3, and Prdm1 (Blimp1)^49^-^56^, TFs that are critical regulators of Th1, Th17, Treg, and B cell differentiation and function.

Notably, our target-based TF enrichment map highlighted MAF (c-Maf) as a prominent TF node within the network associated with the miR-365a-5p target sites. This is mechanistically relevant because c-MAF is a key transcriptional regulator of IL-10 and broader immunoregulatory gene programs across multiple CD4^+^ T-cell contexts, including IL-27 driven induction of IL-10 producing regulatory phenotypes and IL-10 regulation during effector polarization^21^,^22^,^57^. Accordingly, these bioinformatic predictions provided a rationale to test whether selectively enriched EVs with miR-365a-5p is sufficient to reproduce key IL-10/Treg-skewing effects.

In parallel, prior work has shown that miR-365 is a negative regulator of IL-6 expression via direct targeting of the IL-6 3′UTR^58^. This is notable given IL-6's established role in favoring pro-inflammatory CD4^+^ T-cell programs and antagonizing regulatory differentiation, including impaired Foxp3^+^ T reg induction and promotion of Th17 polarization^59^,^60^. Thus, enrichment of miR-365a-5p in glycolytic EVs is consistent with a shift away from IL-6-supported effector programs toward an IL-10-dominant regulatory milieu, while recognizing that EV-mediated effects likely reflect combinatorial contributions from multiple cargo components.

To reduce arbitrariness in highlighting individual miRNAs from a large differentially expressed set, we prioritized candidates that were (i) significantly upregulated, (ii) highly abundant across EVs-UC-MSC_glyco_ samples, and (iii) showed predicted convergence on immune-relevant transcription factor/pathway annotations in our enrichment framework. Based on these quantitative and functional criteria, miR-365a-5p and miR-4478 were selected as representative candidates for further interpretation. This prioritization is supported by previous evidence that miRNAs delivered by MSC-derived small extracellular vesicles can alter gene expression in recipient cells and contribute to tissue repair and anti-inflammatory effects^61^. Notably, miR-365a-5p has been reported as an abundant miRNA in human UC-MSC-derived exosomes, with functional validation indicating direct regulation of target transcripts (e.g., SAV1) and downstream pathway modulation using complementary approaches such as RNA-seq/qPCR and luciferase reporter assays^62^. In contrast, published functional data in rheumatoid arthritis fibroblast-like synoviocytes suggest that miR-4478 can promote pathogenic synoviocyte activation, indicating that its role may be context- and cell type–dependent and warrants targeted validation. While these external data increase biological plausibility, the specific contribution of miR-365a-5p and miR-4478 to the immunomodulatory phenotype in our arthritis models remains to be directly tested by functional perturbation.

Modulation of these transcriptional programs may be consistent with the enhanced immunoregulatory phenotype observed with UC-MSC-derived EVs; however, whether specific miRNAs are necessary and/or sufficient to drive these effects remains to be determined. Notably, EV-mediated effects are not necessarily dependent on intravesicular miRNA transfer, as EV surface-exposed molecules and other cargo components may also trigger immunomodulatory signaling in recipient cells^63^. Thus, the enhanced activity of EVs-UC-MSC_glyco_ likely reflects combined contributions from multiple EV constituents.

While we identify a distinct EV miRNA signature associated with glycolytic reprogramming and performed target prediction analyses, our study does not establish causality because we did not perform gain or loss of functions experiments to test whether specific miRNAs are necessary and/or sufficient for the observed effects. In addition, we did not perform complementary EV proteomic, lipidomic, or metabolomics profiling, including surface-exposed protein-enzymes, which may also contribute to immunomodulation. Future studies integrating EV multi-omics with functional perturbation will be required to define the key EV determinants underlying the enhanced activity of EVs-UC-MSC_glyco_.

## 5. Conclusion

In summary, our findings demonstrate that glycolytically reprogrammed UC-MSC-derived EVs possess enhanced therapeutic potential for autoimmune inflammation by modulating effectors and regulatory T and B cell responses. This effect is likely mediated by their miRNA cargo, which targets key transcription factors involved in immune regulation. While the precise roles of individual miRNAs remain to be defined, their characterization may guide the development of next-generation, miRNA-based acellular therapies for rheumatoid arthritis and related autoimmune disorders.

## Supplementary Material

Supplementary materials and methods, figures.

## Data Availability

All data relevant to this study are included in the manuscript and its supplementary materials. The EV miRNA-seq datasets supporting the conclusions of this article are available in zenodo records (https://zenodo.org/records/18521191). The analysis code supporting the conclusions of this article are available on GitHub (https://github.com/cfarkas/miRNA_exosomes/tree/main). Additional information will be provided by the corresponding authors upon reasonable request.
