# Supplementary material for: Extracellular vesicles from glycolytic mesenchymal stromal cells restrain arthritis progression via IL-10–Producing T and B cells
Source: Theranostics. 2026 May 11;16(12):6713–31. doi: 10.7150/thno.123898 (PMC13231966; doi:10.7150/thno.123898)
Supplement: Supplementary file 1 — Supplementary materials and methods, figures. [file thnov16p6713s1.pdf]

SUPLEMMENTARY MATERIALS

SUPPLEMENTARY FIGURES:

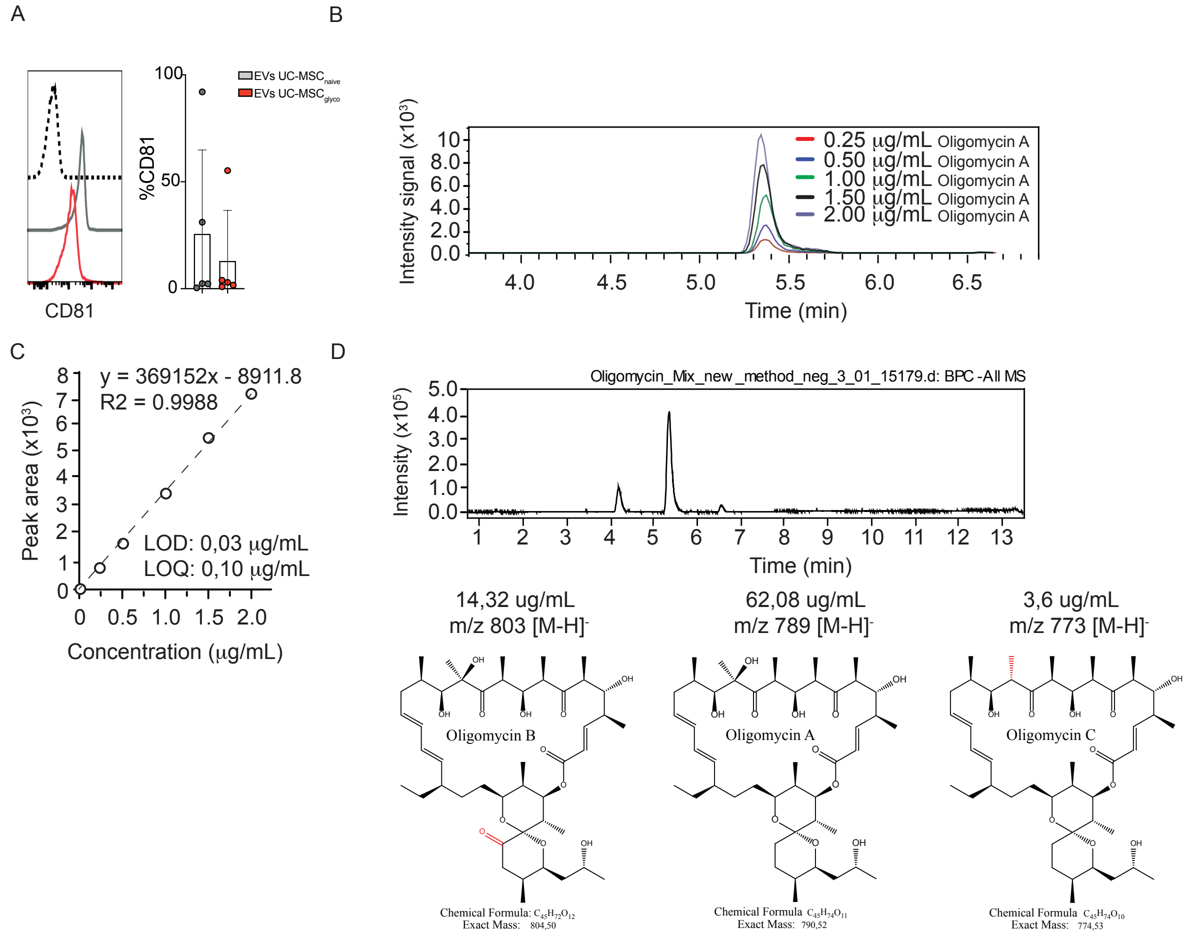

Supplementary Figure 1. EVs characterization and oligomycin detection standardization. (A) Flow cytometry analysis confirming CD81 expression on EVs derived from naïve UC-MSCs (EVs-UC-MSC$_{naive}$) (grey) and glycolytic UC-MSCs (EVs-UC-MSC$_{glyco}$) (red). **(B–D)** Mass spectrometry-based standardization for oligomycin detection. **(B)** Detection at varying concentrations to establish detection limits. **(C)** Mathematical extrapolation for defining threshold values. **(D)** Mass spectrometry analysis showing the presence of specific oligomycin isoforms, each with distinct concentration and m/z (mass-to-charge) ratios.

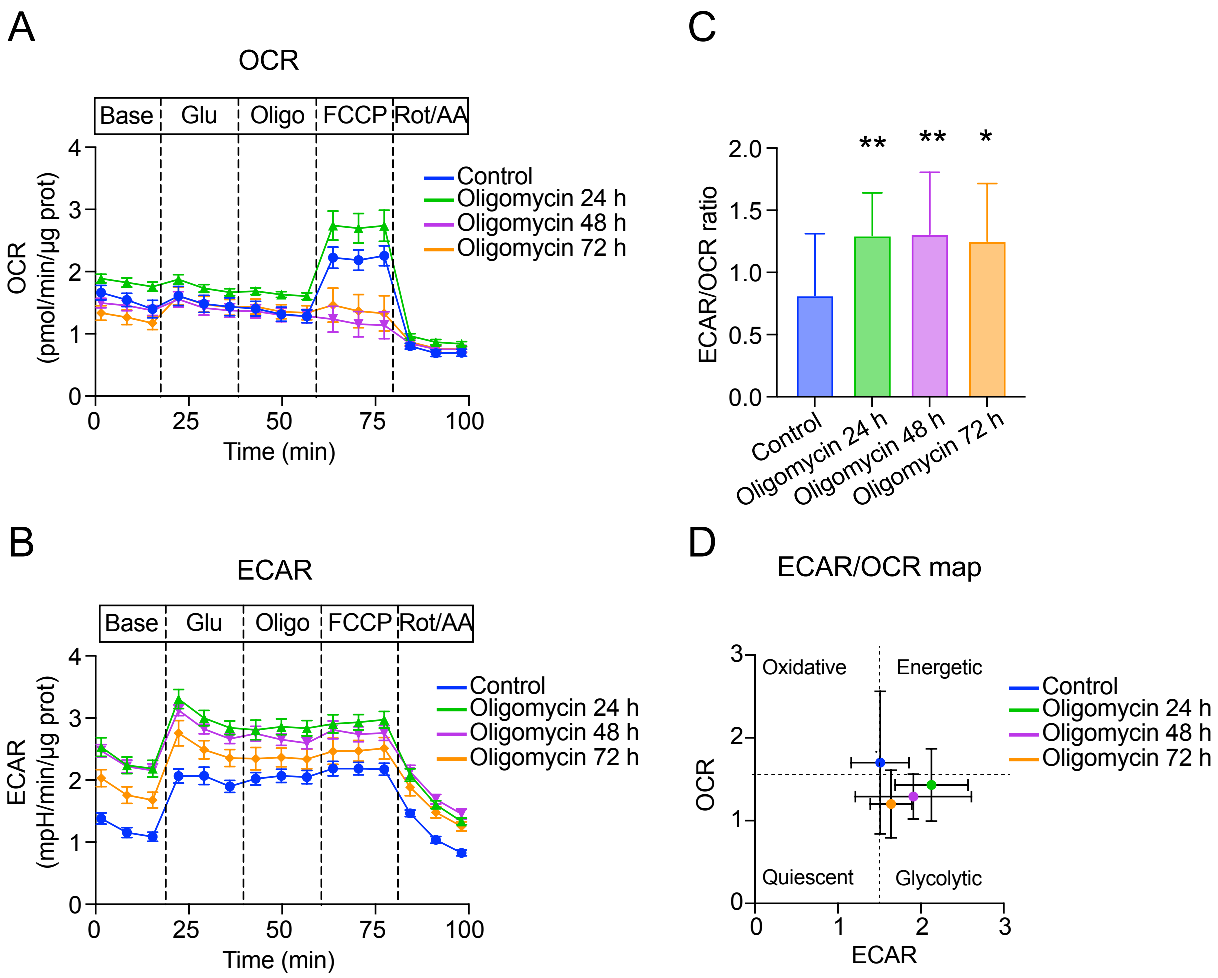

Supplementary Figure 2. Bioenergetic profile shift of UC-MSCs following oligomycin treatment. Human umbilical cord–derived mesenchymal stromal/stem cells (UC-hMSCs) were analyzed using a Seahorse XF96 pro analyzer to quantify **(A)** oxygen consumption rate (OCR; pmol/min/µg protein) and **(B)** extracellular acidification rate (ECAR; mpH/min/µg protein) over the course of the assay under control conditions (blue) or after oligomycin treatment for 24 h (green), 48 h (purple), or 72 h (orange). Measurements were recorded at baseline and after sequential injections of 25 mM glucose (Glu), 1 µM oligomycin (Oligo), 1 µM FCCP, and 0,5 µM rotenone/antimycin A (Rot/AA), as indicated. **(C)** ECAR/OCR ratio as a surrogate of the glycolytic-to-oxidative balance. **(D)** The ECAR/OCR energy map illustrates the relative shift toward a glycolytic phenotype.

Data are shown as mean ± SD from three independent UC-MSC donors (n = 3). Group comparisons were performed using one-way ANOVA with Tukey's post hoc, and significance was set at $p < 0.05$. *$p < 0.05$, **$p < 0.01$.

Supplementary Figure 3

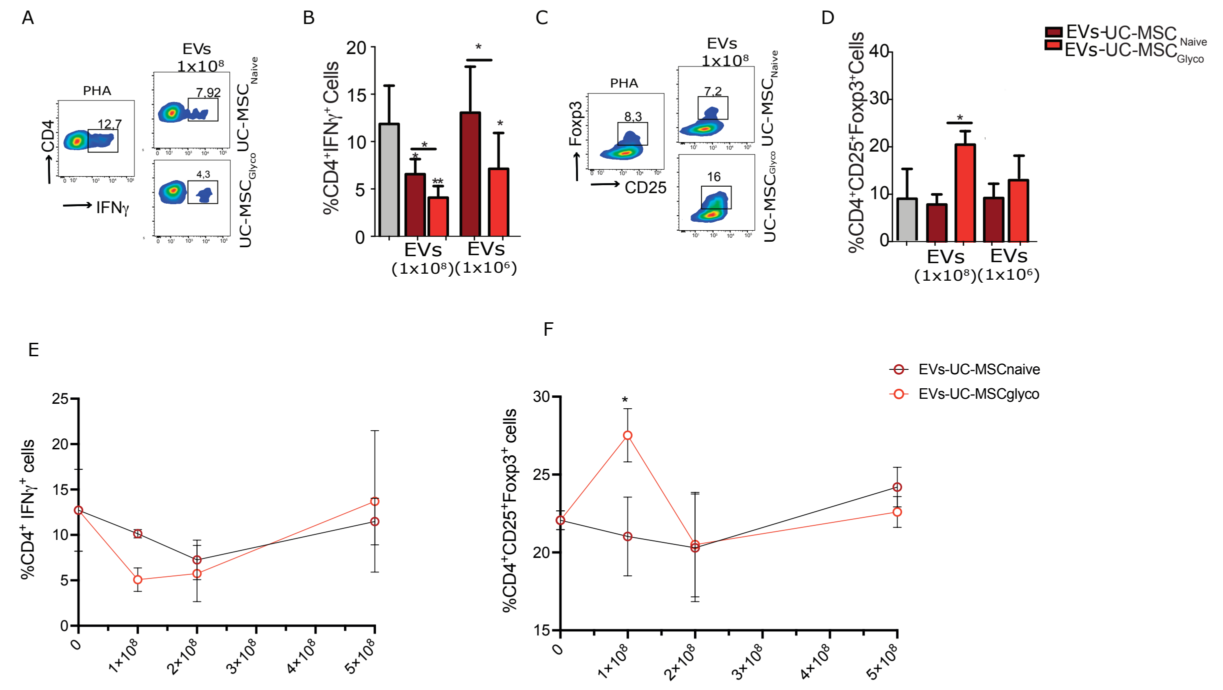

Supplementary Figure 3. Dose-dependent effects of UC-MSC–derived EVs on $CD4^+$ T-cell activation and regulatory phenotype

(A) Representative flow cytometry dot plots and quantification of proinflammatory Th1 (IFN-γ+) cells **(B).** PBMCs stimulated with PHA, treated or not with either EVs-UC-MSC_{naive} or EVs-UC-MSC_{glyco} with two doses of EVs (1×10^6 and 1×10^8 EVs). **(C)** Representative flow cytometry dot plots and quantification of $CD25^+$ $Foxp3^+$ regulatory T cells (Tregs) **(D)** under the same treatment conditions. Gray bars: stimulated PBMCs alone; brown: treated with EVs-UC-MSC_{naive} red: treated with EVs-UC-$MSC_{glyco,}$ **(E, F)** Quantification of proinflammatory Th1 (IFN-γ^+) cells **(E)** and $CD25^+$ $Foxp3^+$ regulatory T cells (Tregs) **(F)**. Memory T-$CD4^+$ T cells after activation with CD3/CD28 beads, treated or not with either EVs-UC-MSC_{naive} or

EVs-UC-MSC$_{glyco}$ following treatment with increasing doses of UC-MSC–derived EVs (1×10^8 to 5×10^8). Data are presented as mean ± SD from n = 4 independent experiments. Statistical analyses were performed using one-way ANOVA followed by Tukey's multiple-comparison test. *$p < 0.05$, **$p < 0.01$.

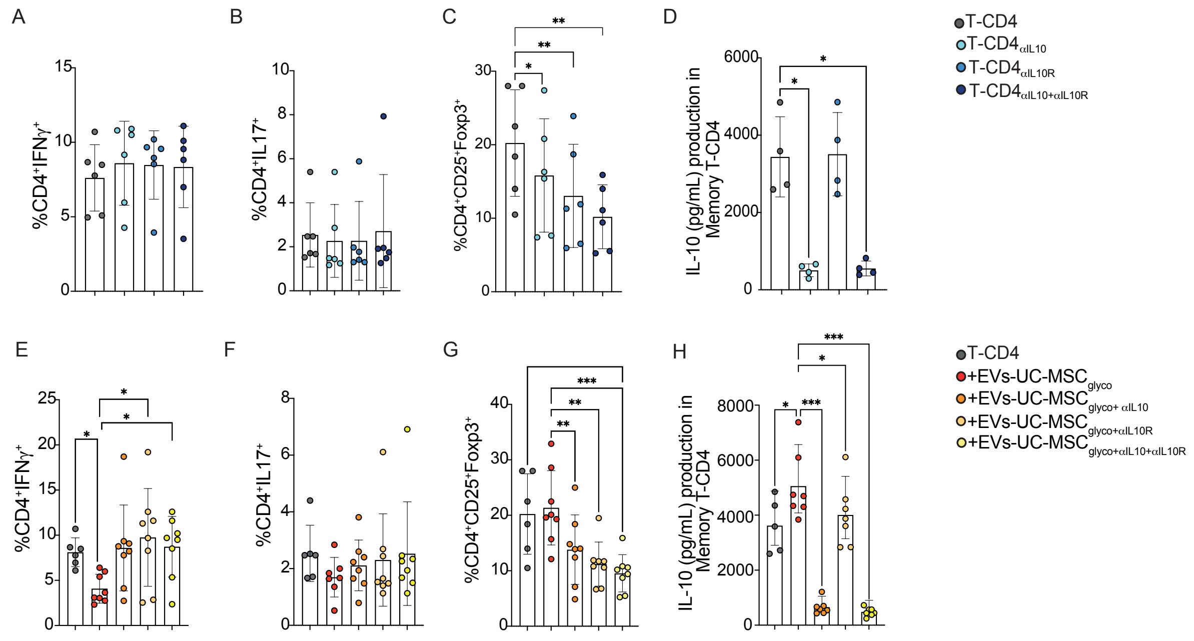

Supplementary Figure 4. IL-10/IL-10R blockade partially reverses EV-mediated modulation of CD4$^+$ memory T-cell responses

(A–C) Quantification of proinflammatory Th1 (IFN-γ$^+$) cells **(A)**, Th17 (IL-17$^+$) **(B)** and CD25$^+$ Foxp3$^+$ regulatory T cells (Tregs) **(C).** Memory T-CD4$^+$ T cells after activation with CD3/CD28 beads, in the presence or absence of neutralizing antibodies against IL-10 (αIL-10), IL-10 receptor (αIL-10R), or their combination. **(D)** IL-10 production measured in supernatants of CD4$^+$ memory T cells cultured under the conditions described in (A–C). **(E–G)** Quantification of proinflammatory Th1 (IFN-γ$^+$) cells **(E)**, Th17 (IL-17$^+$) **(F)** and CD25$^+$ Foxp3$^+$ regulatory T cells (Tregs) **(G).** Memory T-CD4$^+$ T cells after activation with CD3/CD28 beads treated with EVs-UC-MSC$_{glyco}$ in the presence or absence of neutralizing antibodies against IL-10 (αIL-10), IL-10 receptor (αIL-

10R), or their combination. **(H)** IL-10 production measured in supernatants of $CD4^+$ memory T cells cultured under the conditions described in (E-G). Showing the reversal of EV-mediated immunomodulatory effects upon IL-10 and/or IL-10R neutralization. Data are shown as mean ± SD from n = 5 independent experiments. Statistical significance was determined using one-way ANOVA with Tukey's post hoc test. *p < 0.05, **p < 0.01, ****p* < 0.001, *****p* < 0.0001

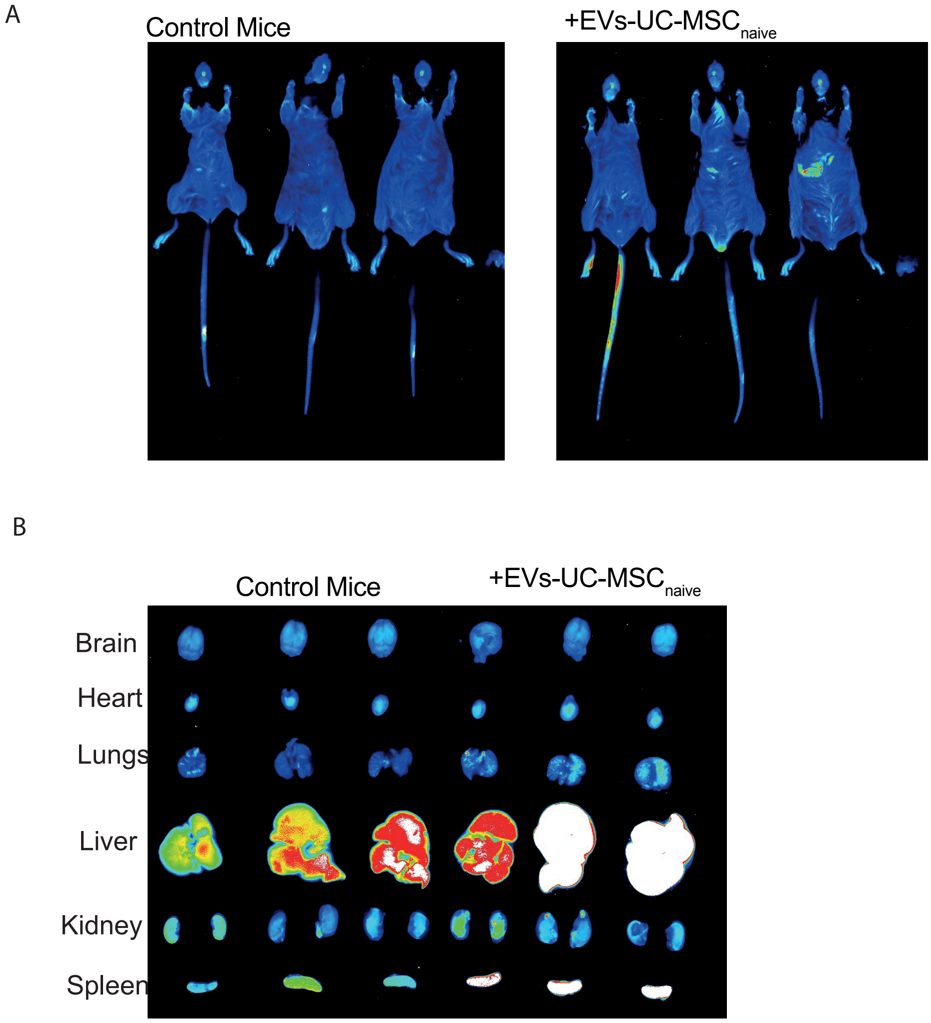

Supplementary Figure 5. Biodistribution of intravenous injection of DiR-labeled EVs in mice. (A) Representative ex vivo fluorescence images of major organs (liver, spleen, lung, kidney, heart, and brain) collected at 6h following intravenous injection of DiR-labeled extracellular vesicles (EVs) in

mice. **(B)** Fluorescence signal intensity indicates the relative accumulation of EVs in each tissue, with higher signal detected in liver and spleen. Images are representative of n = 3 mice per group.

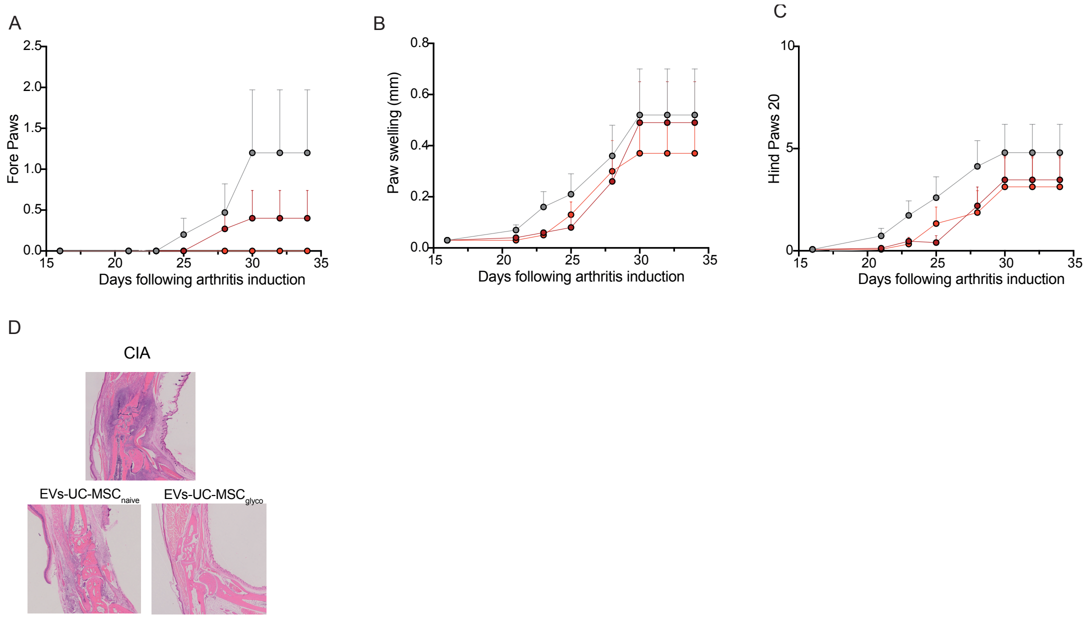

Supplementary Figure 6. Inhibition of inflammation as measured by the Fore Paws (A), Paw Swelling **(B)** and the Hind Paw 20 **(C)** score analysis in the CIA murine model. **(D)** Representative images of ankle joint using HE stain from a CIA mouse group (top), EVs-UC-MSC_{naive} -treated mouse group (left) and EVs-UC-MSC_{glyco} -treated mouse group (right). Statistical analysis used a one-way ANOVA followed by Tukey's multiple comparison test, with at least n=12 biological replicates and $*p < 0.05$, $**p < 0.01$, $***p < 0.001$, $****p < 0.0001$.

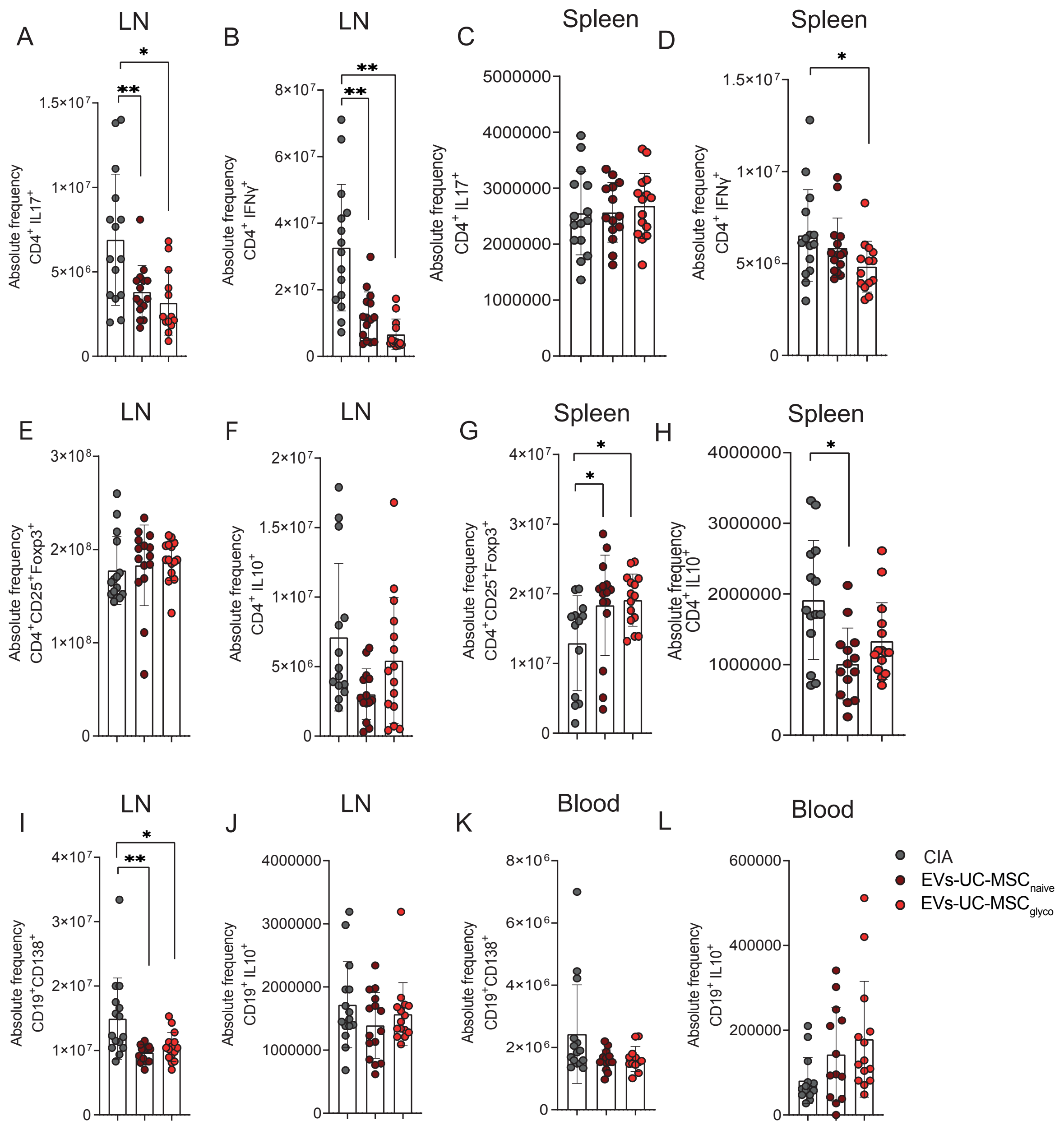

Supplementary Figure 7. EVs-UC-MSC$_{glyco}$ modulate T lymphocyte populations in CIA mice. Absolute frequency analysis of T cell subsets in collagen-induced arthritis (CIA) mice (gray), and mice treated with EVs-UC-MSC$_{naive}$ (brown) or EVs-UC-MSC$_{glyco}$ (red). **(A-D)** Absolute frequency of proinflammatory CD4^{+}IL-17^{+} Th17 cells in the lymph nodes **(A)** and spleen **(C)** Absolute frequency of CD4^{+}IFN-γ^{+} Th1 cells in the lymph nodes **(B)** and spleen **(D)**. **(E-H)** Absolute frequency of CD4^{+}CD25^{+} Foxp3^{+} regulatory T cells (Tregs) in the lymph nodes **(E)** and spleen **(G)**. Absolute frequency of CD4^{+}IL-10^{+} type 1

regulatory T cells (Tr1) in the lymph nodes **(F)** and spleen **(H)**. **(I-L)** Frequency of CD19+CD138+ plasma cells in the blood **(K)** lymph nodes **(I)** and CD19+IL10+ Breg cells in blood **(L)** and in lymph nodes **(J)** One-way ANOVA with Tukey's multiple comparison test was used for statistical analysis, with at least n=12 biological replicates and $*p < 0.05$, $**p < 0.01$.

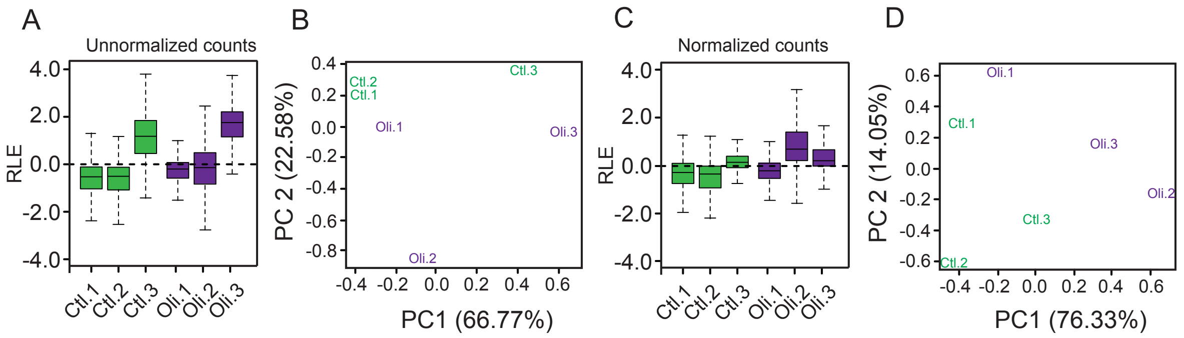

Supplementary Figure 8. Principal component analysis (PCA) of EVs-UC-MSC$_{glyco}$ or EVs-UC-MSC$_{naive}$. Characterization of PC1 and PC2 components based on RNA-seq data from EVs isolated from either glycolytic or naïve UC-MSCs (B and C). Read count data were normalized using Relative Log Expression (RLE) normalization across the three samples to ensure comparability (A and B).

SUPPLEMENTARY MATERIALS AND METHODS

Transmission Electronic Microscopy (TEM). The particle suspension is dropped as droplets onto electron microscopy screens, a copper-coated grid, which is adsorbed for 20 min. The grids were then fixed with 1% glutaraldehyde and washed with deionized water. Subsequently, the grids were counterstained with aqueous uranyl acetate for 10 min and washed again with distilled water, finally, and allowed to dry in the open air. The images were acquired and observed

on the Talos F200C G2 transmission electron microscope, Ceta 16M CMOS, camera pixel size 14μm x 14μm, 16bit, resolution 0.3nm.

Internalization analysis by confocal microscopy and FACS. Isolated vesicles were labeled with the lipophilic red fluorescent dye PKH26 (sigma) following the manufacturer's instructions and then subjected to ultracentrifugation (100,000 x g) for 1 hour to wash the vesicles and remove excess dye. The labeled vesicles were contacted with naïve CD4+ T cells or purified memory CD4+ T cells using the isolation kit, EasySep™ Human Naïve CD4+ T Cell Isolation Kit (Cat. #19555) and EasySep™ Human Memory CD4+ T Cell Enrichment Kit (Cat. #19157) respectively and activated with Dynabeads human T-activator CD3/CD28 beads (Invitrogen), supplemented with IL-2 [250u/ml] for memory T cells. Vesicle internalization was assessed by confocal microscopy (Zeiss LSM 780-NLO) and flow cytometry (BD, pharmingen). Lymphocytes were evaluated after 24 hours of culture with the labeled vesicles. For microscopic analysis, the cells were washed with PBS and fixed with 4% formaldehyde (Sigma – Aldrich), then blocked with PBS 5% BSA for 20 minutes and labeled with the anti-CD3, anti-CD4 fluorescent conjugated antibody. The antibodies were diluted in PBS 1% BSA and incubated for 40 min. Nuclei were counterstained with DAPI. For FACS analysis, cells were obtained and evaluated by flow cytometry analysis using the Cytek Aurora cell analyzer.

Western blot analysis. Western blot analysis to evaluate identity and purity of sEV according to MISEV (15) was conducted following the protocols as we previously described. Briefly, a volume of 50 μL of isolated sEV samples as well as 2 μg of cell lysate, both type of samples derived from control and oligomycin-treated cells, were loaded per lane. An 8 to 260 kDa fluorescent protein ladder was used to determine protein size (LI-COR Biosciences, Cat. #928-60000, 1 to 2

μL per lane). Primary antibodies targeting Syntenin-1 (endosomal origin extracellular vesicles marker; Novus Biologicals, Cat. #NBP2-76873, 1:1000 dilution), Flotillin-1 (extracellular vesicles membrane marker; Abcam, Cat. #ab133497, 1:2000 dilution), Calnexin (endoplasmic reticulum marker; Abcam Cat. #ab22595, 1:2000 dilution) and TOMM20 (mitochondria marker; Novus Biologicals, Cat. #NBP2-67501, 1:1000 dilution) were used as identity and purity markers. Goat anti-Rabbit Alexa Fluor Plus 800 (Invitrogen, Cat. #A32735, 1:25000 dilution) was used as secondary antibody. Digital images of the blot were captured using a LI-COR Odyssey CLx imaging system (LI-COR Biosciences).

Detection of oligomycin traces in glycolytic EVs-UC-MSC. Sample preparation and metabolome extraction were performed according to a previously described protocol with modifications(26). Briefly previously frozen samples were thawed at room temperature and then transferred to filter tubes (Amicon Ultra-0.5 Centrifugal Filter Unit) to remove PBS. The samples were filtered at 15,000 g for 10 minutes at 4°C. Once the PBS was discarded, 600μL of extraction solvent (Methyl tertvutyl ether (MTBE)/Methanol/Water, 5:3:2, v/v) at 4°C was added to the same tube. The entire content, along with the ternary mixture of solvents, was transferred to another tube, where a 3 mm zirconium bead (POWTEQ) was added for extraction by agitation at 30 Hz for one minute using a mixer mill (MM400 RETSCH). After this step, 400μL of H_2O and 400μL of MTBE were added to perform a liquid-liquid extraction, allowing the simultaneous collection of the lipidic and polar phases (Folch-type extraction). After agitation at 20 Hz for 1 min and centrifugation at 13,300 g for 5 min, both the polar and apolar phases were sharply defined, collected and dried for 5 hours and 2 hours, respectively, at 30°C in an Eppendorf Plus concentrator (drying mode V – HV). Before LC-MS analysis,

polar extracts were reconstituted in 800 µL of 80% methanol (in Milli-Q water, v/v) at 4°C, agitated for 1 min at 30 Hz and centrifuged for 10 min at 13,000 rpm. Then, 200µL of supernatants were transferred to inserts placed in vials and stored in the liquid chromatography autosampler at 4°C till analysis.

LC-MS analysis. The sample analysis was carried out using an ultra-high-performance liquid chromatography (UHPLC) system coupled to a high-resolution mass spectrometer through an atmospheric pressure electrospray ionization (API-ESI) interface and with a hybrid quadrupole time-of-flight mass analyzer (HR/Q-TOF/MS, Compact Bruker Daltonik GmbH, Bremen, Germany). Chromatographic separation was performed on a UHPLC Kinetex C18 column (100 × 3.0 mm, 1.7 µm; Phenomenex) using a Security Guard Ultra Cartridge UHPLC C18 (3.0 mm) pre-column, maintained at 30°C. The mobile phase consisted of solvent A (Milli-Q water with 0.1% formic acid, v/v) and solvent B (acetonitrile hypergrade with 0.1% formic acid, v/v) at a flow rate of 0.4 mL min^{-1}. The elution was performed using a linear gradient starting with a 2.0 min equilibration time at 70% B, followed by the following program: 0.0 – 10.0 min, 70.0 – 100% B (linear); 10.0 – 14.0 min, 100% B; 14.0 – 14.5 min, 100 – 70% B (linear). The sample injection volume was 5.0µL. For trace quantification of oligomycin, a calibration curve was prepared using the oligomycin A standard in a concentration range of 0.25 mg mL^{-1} - 2 mg mL^{-1} and analyzed using the same method as the used for samples. The calibration curve was used to determine the limits of detection (LOD) and quantification (LOQ) of the method. The mass spectrometer operation was optimized for the detection of oligomycin. It was operated in negative electrospray ionization (ESI) modes, acquiring data in full-scan mode to enhance sensitivity. The MS parameters were set as follows: mass range, 700 – 900 m/z; scan cycle, 1 sec; drying temperature, 200°C; capillary voltage, 3.5 kV; endplate offset, 0.5

kV; desolvation gas flow, 9.0 L min^{-1}; nebulizer pressure, 2.0 Bar. An internal reference solution of sodium formate (10 mM in iso-PrOH/H_2O, 1:1, v/v) was used, for mass calibration. This solution was pumped at a rate of 1μL min^{-1} through a six-port pump, injecting 20 μL into the mass spectrometer before each analysis.

Isolation of human peripheral blood mononuclear cells (PBMCs). Blood samples from healthy donors (men and women, age range 23-35 years old) were obtained after all donors provided written informed consent. PBMCs were obtained from 20 ml of heparinized blood and diluted 1:1 in 1X phosphate-buffered saline (PBS). A layer of diluted blood was placed on the density gradient of Ficoll-Plaque PLUS (Sigma-Aldrich, St.Louis , MO, USA). It was then centrifuged at 400 x g for 30 min. Isolated buffy coat cells were collected and washed twice with PBS solution, centrifuging the cells at the same speed for 15 min. Naïve CD4 cells were then isolated from PBMCs using the EasySep™ Human Naïve CD4+ T Cell Isolation Kit (Cat. #19555) and memory CD4 cells were also isolated from PBMCs using the selection isolation kit. negative EasySep™ Human Memory CD4+ T Cell Enrichment Kit (Cat. #19157).

Immunoglobulin Quantification. Serum samples were collected at different time points after collagen-induced arthritis (CIA) immunization. Anti-bovine type II collagen (bCII) antibodies were measured by isotype-specific ELISA. Plates were coated with bCII (5 μg/mL) overnight at 4 °C, washed, and blocked with PBS containing 2% BSA for 1 h at room temperature. Serum samples were diluted 1:300 and 1:600 for IgG1 and IgG2 detection (BD Pharmingen), and 1:6000 and 1:12000 for total IgG detection(BD Pharmingen). IgG1 and IgG2 were detected using alkaline phosphatase–conjugated secondary antibodies with *p*-nitrophenyl phosphate (pNPP) substrate (Sigma-Aldrich).

Co-culture of PBMCs with EVs-UC-MSC. PBMCs (2,5 x 10^5 cells) were plated in IMDM medium supplemented with 10% fetal bovine serum (FBS) (Hyclone), 1% penicillin/streptomycin (Gibco), 1% L-glutamine (Gibco), 1% MEM non-essential amino acids, 1% pyruvate (Gibco), 0.1% β-mercaptoethanol (Gibco) (full MLR) (Gibco) (full MLR) and stimulated with phytohemagglutinin (PHA) [5ug/ml], PBMCs stimulated with PHA and stained with cell trace violet (CTV) (20 minutes), to evaluate their proliferation rate, were co-cultured with the same volume of PBS, or EVs-UC-MSC_{naive} or EVs-UC-MSC_{glyco} (1 x 10^8 particles) for 5 days. The percentage of proliferation of CD4+ T cells was evaluated, and the percentage of CD4+ IFNγ + Th1 cells and CD4+CD25+ Foxp3+ Tregs.

Removal of Unwanted Variation (RUVSeq). Potential technical biases and batch effects were minimized using RUVSeq (version 1.28.0)(27). A generalized linear model (GLM) was first fitted to the raw counts, and its residuals were passed to the RUVr function to estimate empirical factors of unwanted variation (k=1). This approach generated "empirically normalized" counts. The success of the correction was confirmed by comparing PCA and RLE plots before and after normalization.

Presence/Absence Heatmaps for Transcription Factors and Immune-Related Terms. To determine whether specific miRNAs converge on particular transcription factor (TF) families or immune pathways, two presence/absence heatmaps were created, a TF Heatmap consisting in a custom dictionary of TF synonyms (e.g., FOXP3, GATA3, TBX21) used to detect TF-related annotations. Each miRNA was labeled "present" if its target annotations matched any known TF target genes with statistical significance in GO analyses (p-value>0.01). Also, an Immune Heatmap was created using a list of immune-related keywords (e.g.,

“lymphocyte activation,” “innate immune response”) was similarly applied, marking each miRNA targets that showed enrichment for these immune terms.
